# Global assessment of spatiotemporal variability of wet, normal and dry conditions using multiscale entropy-based approach

**DOI:** 10.1038/s41598-022-13830-w

**Published:** 2022-06-13

**Authors:** Vijay Sreeparvathy, V. V. Srinivas

**Affiliations:** 1 Department of Civil Engineering, Indian Institute of Science, Bangalore, 560 012 India; 2 Interdisciplinary Centre for Water Research (ICWaR), Indian Institute of Science, Bangalore, 560012 India; 3 Divecha Centre for Climate Change, Indian Institute of Science, Bangalore, 560 012 India

**Keywords:** Hydrology, Climate-change impacts, Climate-change mitigation

## Abstract

In recent decades, human-induced climate change has caused a worldwide increase in the frequency/intensity/duration of extreme events, resulting in enormous disruptions to life and property. Hence, a comprehensive understanding of global-scale spatiotemporal trends and variability of extreme events at different intensity levels (e.g., moderate/severe/extreme) and durations (e.g., short-term/long-term) of normal, dry and wet conditions is essential in predicting/forecasting/mitigating future extreme events. This article analyses these aspects using estimates of a non-stationary standardized precipitation evapotranspiration index corresponding to different accumulation periods for 0.5° resolution CRU grids at globe-scale. Results are analyzed with respect to changes in land-use/landcover and geographic/location indicators (latitude, longitude, elevation) at different time scales (decadal/annual/seasonal/monthly) for each continent. The analysis showed an (i) increasing trend in the frequency/count of both dry and wet conditions and variability of dry conditions, and (ii) contrasting (decreasing) trend in the variability of wet conditions, possibly due to climate change-induced variations in atmospheric circulations. Globally, the highest variability in the wet and dry conditions is found during the Northern hemisphere's winter season. The decadal-scale analysis showed that change in variability in dry and wet conditions has been predominant since the 1930s and 1950s, respectively and is found to be increasing in recent decades.

## Introduction

Environmental extreme events (e.g., heat waves, droughts, snowmelts, floods) occur as a long- or short-term phenomenon. They produce a complex web of direct and indirect impacts on various (e.g., economic, environmental and social) sectors of life. In recent decades, globally, the average annual count of people affected by drought and flood events is estimated to be ~ 55 million and ~ 1.4 billion, respectively^1,2^. Early forecast and warning of the possible extreme events could facilitate better preparation, possible evacuation and marshalling of emergency systems to mitigate environmental impacts. However, prediction/forecasting of extreme events has become difficult. It is due to the (i) intensification of the hydrologic cycle and spatiotemporal variability of atmospheric circulation patterns associated with changing climate, land-use/landcover (LULC), and anthropogenic activities, and (ii) complex nonlinear interactions of the hydroclimatic variables. Hence, an in-depth understanding of the variability and trends of extreme events is crucial for effective monitoring and prediction of possible future extreme events and for proposing appropriate adaptation/mitigation strategies.

Several drought indices have been developed to detect/monitor, and quantify characteristics (e.g., frequency, severity, and spatial extent) of dry/wet conditions in different regions^3,4^. Conventional drought indices (e.g., Standardized Precipitation Index^5^, Standardized Precipitation Evapotranspiration Index^6^ (SPEI), Palmer Drought Severity Index^7^, Standardized Hydrologic Index^8^) are determined assuming stationarity^9^ in hydrometeorological time-series. The estimation involves fitting a stationary distribution to historical timeseries of reference variables corresponding to different accumulation periods (APs), considering parameters as time-invariant. However, the timeseries could exhibit non-stationarity due to changing climatic conditions^10–12^. In such scenarios, the characteristics of dry/wet events discerned using indices determined by stationary analysis may not be accurate. To address this, a new non-stationary version of the SPEI (NSPEI) is considered in this article. It accounts for non-stationarity in precipitation and evapotranspiration by fitting a non-stationary time-varying distribution to the drought reference variable. Hence it is deemed effective to identify and monitor wet/dry events (droughts, floods) of different intensity levels corresponding to various accumulation periods (APs) (1, 3, 6, 12 and 24 months) for changing climatic conditions. The time scale (e.g., 1 month, 24 months) over which the deficits/excesses of various hydrologic variables (e.g., precipitation) accumulate becomes extremely important to separate different types of water-excess/deficit conditions and assess their severity. For example, in the water-deficit context, APs of 1-month, 3 to 6 months, and greater than 12-months are predominantly relevant to meteorological, agricultural, hydrological, and socio-economical droughts, respectively^13–16^. Furthermore, in the water-excess context (i.e., wetter-than-normal conditions), analysis at different APs enhances the capacity to estimate different soil antecedent conditions. Short APs (1, 3 and 6 months) help quantify the state of surface soil moisture, which is of direct significance for agriculture. In contrast, longer APs (e.g., 12 to 72 months) indicate the state of subsoil moisture and other surface/subsurface water resources. Therefore, the joint consideration of different APs can satisfactorily explain the antecedent conditions and risk associated with likely flood/drought events^17–19^.

Variability of extreme events can be quantified as the lack of uniformity over different spatiotemporal scales. Spatial variability characterizes the uncertainty of extreme events over multiple geographic locations, whereas temporal variability quantifies the uncertainty across different time scales. Attempts have been made in previous studies to assess the variability of droughts in terms of the magnitude of different drought indices and the associated hydro-climatic predictor variables/covariates^20–25^. Despite these studies, quantifying the variability of drought intensity and duration continues to be a problem. Even though various studies have tried to quantify the variability of hydrologic variables^26–28^, to the best of our knowledge, none of the previous studies has quantified the global-scale spatiotemporal variability and trends in variability for various intensity levels of dry/wet conditions at multiple timescales and APs. Hence to address these issues, a multiscale (decadal, inter- and intra- annual) entropy-based approach is considered to quantify the uncertainty associated with the normal condition and different intensity levels (moderate, severe, and extreme) of dry/wet conditions. The main advantage of using entropy-based measures to quantify the uncertainty associated with a timeseries (e.g., NSPEI) is that they do not make prior assumptions on the data's probability distribution or statistical properties^29^. Hence, they are deemed appropriate for use in analysis with NSPEI. However, the non-normalized uncertainty estimates corresponding to timeseries of different time scales and data lengths cannot be readily compared^27^. Moreover, even when the entropy estimates have the same value at different geographical locations, the uncertainty associated with dry/wet conditions may differ across the locations depending on the regional climatic characteristics and LULC patterns. Hence to address these issues, a standardized variability index (SVI) with a finite range of [0,1] is considered in this study to quantify the variability of normal and different intensity levels of dry and wet conditions for various APs (1-,3-,6-,12- and 24-months). The variability was also evaluated with respect to changes in LULC patterns and location indicators (latitude, longitude, and elevation) for different continents (except Antarctica). Furthermore, analysis is carried out to identify trends in intra-annual variability and frequency of wet/normal/dry conditions.

The study's outcomes can enhance understanding of spatiotemporal patterns of the dry and wet conditions and their variability at the global scale. This could help identify regions and LULC classes across different continents that are most vulnerable to variations in dry/wet conditions and devise effective mitigation strategies. Moreover, the foregoing information can be used by policymakers to devise effective water management (e.g., irrigation practices) and drought/flood risk mitigation plans^30,31^ (e.g., setting-up early warning systems).

## Methods and data

This section provides the methodology considered in this study to assess the spatiotemporal variability of dry and wet conditions for different intensity levels and normal conditions. Flowcharts showing the complete methodology and data considered in this study are provided in Fig. S2.

### Estimation of NSPEI

Estimates of drought intensity corresponding to different APs were obtained using a new time-dependent index, NSPEI. It is defined as analogous to the conventional SPEI. However, it considers a non-stationary GEV (generalized extreme value) distribution with a time-varying location parameter in lieu of stationary GEV or log-logistic distributions used in the case of conventional SPEI. The GEV distribution was considered in this study, as it showed the best goodness of fit value across different APs and proven consistency across multiple datasets and goodness of fit measures^13,32^.

Let $P_{\upsilon ,\tau }$ and ${\text{PET}}_{\upsilon ,\tau }$ denote the precipitation and potential evapotranspiration corresponding to month $\tau$ (= 1,…,12) in the year $\upsilon$ (= 1,…, *N*). The water surplus or deficit (i.e., climate water balance) corresponding to the AP of *M* months is calculated at a month $\tau$ in the year $\upsilon$ as,

1 $$\begin{aligned} & d_{\upsilon ,\tau }^{M} = \sum\limits_{t = 13 - M + \tau }^{12} {\left( {P_{\upsilon - 1,t} - {\text{PET}}_{\upsilon - 1,t} } \right)} { + }\sum\limits_{t = 1}^{\tau } {\left( {P_{\upsilon ,t} - {\text{PET}}_{\upsilon ,t} } \right)} {\text{ if }}\tau < M \\ & d_{\upsilon ,\tau }^{M} = \sum\limits_{t = \tau - M + 1}^{\tau } {\left( {P_{\upsilon ,t} - {\text{PET}}_{\upsilon ,t} } \right)} {\text{ if }}\tau \ge M \\ \end{aligned}$$

The time series obtained for $d_{\upsilon ,\tau }^{M}$ was subdivided into subseries $d_{\tau }^{M}$ corresponding to each month $\tau$. Then a three-parameter time-varying GEV distribution was fitted to each subseries (i.e., $d_{\tau }^{M} \sim {\text{GEV}}\left( {\xi_{\tau } \left( t \right),\alpha_{\tau } ,k_{\tau } } \right)$), where $\xi_{\tau }$, $\alpha_{\tau }$ and $k_{\tau }$ are the location, scale and shape parameters for the month $\tau$. A *q*-th degree polynomial was fitted to the location parameter as $\xi_{\tau } \left( t \right) = \beta_{0} + \beta_{1} t + \ldots + \beta_{q} t^{q}$, where $\beta_{1} {, } \ldots {,}\beta_{q}$ are coefficients. The optimal value of *q* was determined using Akaike Information Criterion^33^ (AIC). The goodness of fit of the time-varying GEV distribution was tested using the Filliben correlation coefficient^34^ and analyzing Q-Q plots between the theoretical and observed/empirical quantile estimates^35^. The non-exceedance probability corresponding to $d_{\tau }^{M}$ was then transformed to a standard normal variable (i.e., NSPEI) using the approximate transformation^36^. Based on the NSPEI value, months were classified into seven intensity levels: extreme wet (${\text{NSPEI > }}2$), severe wet (${1}{\text{.5 < NSPEI}} \le {2}$), moderate wet (${\text{1 < NSPEI}} \le {1}{\text{.5}}$), normal ($- 1 < {\text{NSPEI}} \le {1}$), moderate dry ($- 1.5 < {\text{NSPEI}} \le { - 1}$), severe dry ($- 2 < {\text{NSPEI}} \le { - 1}{\text{.5}}$), and extreme dry (${\text{NSPEI}} \le { - 2}$) (Table S2).

### Estimation of the variability of normal, dry and wet conditions

The variability was quantified at different locations across the globe corresponding to different time scales and APs (1, 3, 6, 12 and 24 months) using various entropy measures (i.e., marginal entropy; decadal entropy; and apportionment entropy). The estimates of marginal entropy are presented at monthly and seasonal time scales, whereas those of other measures are presented at only monthly timescale. This is because there were no noticeable differences in the apportionment and decadal entropies of dry/wet conditions at the seasonal timescale. NSPEI corresponding to *M* months AP was referred to as NSPEI-*M* (e.g., NSPEI-1 for 1 month AP).

#### Marginal entropy (ME)^29^.

It is used to quantify the inter-annual variability (i.e., uncertainty) of the frequency (i.e., count) of wet/normal/dry months corresponding to the chosen time window/scale (yearly or seasonal). It is computed as,

2 $$H^{{{\text{ME}}}} = - \sum\limits_{\upsilon = 1}^{{n_{c} = N}} {\frac{{m_{\upsilon } }}{S}{\text{log}}_{2} \left[ {\frac{{m_{\upsilon } }}{S}} \right]} {\text{ , where }}S = \sum\limits_{\upsilon = 1}^{{n_{c} = N}} {m_{\upsilon } }$$

where *N* represents the number of years for which NSPEI estimates were obtained, $m_{\upsilon }$ represents the total number of months in the time window (i.e., year or season of the year) that are identified by NSPEI to be in the specific condition (i.e., normal, moderate/severe/extreme wet or dry condition). Based on the meteorological temperate seasons^37,38^, predominantly, there are four seasons in the northern hemisphere (NH) and southern hemisphere (SH): (i) spring (March–May in NH; September–November in SH), (ii) autumn (September–November in NH; March–May in SH), (iii) summer (June–August in NH; December-February in SH), and (iv) winter (December-February in NH; June–August in SH). The four seasons of the NH were considered as reference for the analysis as majority of the earth's human population (~ 87.0%) and land area (~ 68%) are in the NH.

#### Decadal entropy (DE)^26^.

It quantifies the inter-annual variability of the frequency of wet/normal/dry months in decadal time windows. It is computed as,

3 $$H^{{{\text{DE}}}} \left( i \right) = - \sum\limits_{{\upsilon = \left( {i - 1} \right) \times n_{c} + 1}}^{{i \times n_{c} }} {\frac{{m_{\upsilon } }}{D}{\text{log}}_{2} \left[ {\frac{{m_{\upsilon } }}{D}} \right]} {\text{ where }}D = \sum\limits_{{\upsilon = \left( {i - 1} \right) \times n_{c} + 1}}^{{i \times n_{c} }} {m_{\upsilon } } { ; }i = 1, \ldots ,n_{w} \,$$

where $\, n_{c} { = }10$, $\, n_{w} { = }\left\lfloor {{N \mathord{\left/ {\vphantom {N {10}}} \right. \kern-\nulldelimiterspace} {10}}} \right\rfloor$, $m_{\upsilon }$ is same as that defined in the case of $H^{ME}$ for yearly time window.

#### Apportionment entropy (AE)^26^.

It quantifies the intra-annual variability of the frequency of wet/normal/dry conditions.

4 $$H^{{{\text{AE}}}} \left( \upsilon \right) = - \sum\limits_{\tau = 1}^{{n_{c} = 12}} {\frac{{\dot{n}_{\upsilon ,\tau } }}{{m_{\upsilon } }}{\text{log}}_{2} \left[ {\frac{{\dot{n}_{\upsilon ,\tau } }}{{m_{\upsilon } }}} \right]} \,$$

where $\dot{n}_{\upsilon ,\tau } = 1$ if the month $\tau$ in year $\upsilon$ has specified condition (i.e., normal, moderate/severe/extreme wet or dry condition) and zero otherwise. When $\dot{n}_{\upsilon ,\tau } = 0$, $H^{{{\text{AE}}}} \left( \cdot \right)$ could be considered to be zero, which is consistent with a well-known limit^39^ ($\mathop {Lt}\limits_{x \to 0} x{\text{log}}x = 0$).

The upper bound for the magnitude of entropy depends on the number of data points used for its estimation, which in turn depends on the record length of observations and the size of the time window considered for evaluation. Hence a Standardized Variability Index (SVI) was considered to facilitate the comparison of entropy of NSPEI estimates obtained for different grids/locations corresponding to multiple time scales, APs and intensity levels (i.e., normal, moderate/severe/extreme wet or dry conditions). The use of SVI to quantify the variability of precipitation and to develop homogeneous precipitation regions can be found in previous studies^27,39,40^. The SVI index is defined as,

5 $$SVI = \frac{{H_{{{\text{max}}}} - H}}{{H_{{{\text{max}}}} }}$$

SVI attains values in the range [0,1], where 0 denotes the least variability and 1 refers to the highest variability. The SVI estimates computed using ME, AE and DE estimates are denoted as SVI_ME_, SVI_AE_ and SVI_DE,_ respectively. The value of entropy *H* ranges from 0 to $H_{{{\text{max}}}}$ ($= {\text{log}}_{2} n_{c}$). *H* = 0 corresponds to the situation where there is no uncertainty. On the other hand, $H = H_{{{\text{max}}}}$ denotes the situation where the event of interest is equally likely to occur in each of the time windows considered for analysis, indicating the highest uncertainty. For example, if all months in a year experience moderate dry condition (i.e., uniform distribution scenario), the probability of occurrence of the dry condition is the same for all the months, indicating high uncertainty or low temporal variability of dryness.

The study was conducted over all the continents of the globe (except Antarctica) by analyzing the variability of NSPEI (dry/wet index) estimates obtained corresponding to different APs (i.e., 1, 3, 6, 12, and 24 months). The index was computed considering 0.5° resolution monthly records of precipitation and potential evapotranspiration extracted for 119 years (1901–2019) from the climate research unit (CRU) global database. The estimates were analyzed to discern the condition (wet, normal or dry) of each month and its intensity level (moderate, severe, or extreme) in wet/dry conditions at each grid in the chosen continents. Subsequently, the variability in occurrence and intensity levels of the aforementioned conditions was assessed for each grid corresponding to different APs. The assessments were at (i) inter-annual (-seasonal and -monthly), (ii) intra-annual, and (iii) decadal scales using the entropy measures SVI_ME_, SVI_AE_ and SVI_DE_, respectively. It is to be noted that the drought/wetness uncertainty analysis may have limited practical implications for deserts and hyper-arid regions. However, it may provide valuable information for the physical understanding of climate change impact in these regions. Hence, the drought/wetness uncertainty analysis is performed on the entire global land area^41^, unlike other global studies that excluded hyper-arid and deserted regions^42,43^. Mann–Kendall (MK) trend test^44,45^ was considered to identify the trend in annual estimates of (i) standardized variability index SVI_AE_ and (ii) monthly frequency/count of normal, wet and dry conditions at various intensity levels and APs at 0.5° resolution precipitation grids covering the globe.

The spatiotemporal variability of wet, normal and dry conditions across different LULC classes and ranges of elevation, latitude and longitude was assessed in each continent, corresponding to different APs and timescales. The LULC and elevation data of the continents were extracted from 100 m resolution Copernicus Global Land Services Land Cover (CGLS-LC, 2015) map and 30 m resolution Shuttle Radar Topography Mission (SRTM) Digital Elevation Model (SRTM-DEM), respectively. The overall accuracy of the CGLS-LC discrete global LC map is 80.6 ± 0.7% at a 95% confidence level. The land cover classes namely forest, bare/sparse vegetation, snow/ice and permanent water are mapped with high accuracy (> 85%). Whereas herbaceous vegetation, croplands and urban LC are at moderate accuracies (65%-85%), and herbaceous wetlands, lichen/mosses, and shrubs have lower accuracies (< 65%). The detailed continent wise spatial uncertainty of different LULC classes could be found in Tsendbazar et al. (2020)^46^.

The seasonal and monthly scale estimates of SVI_ME_ obtained corresponding to various intensity levels (moderate, severe and extreme) of wet and dry conditions and normal conditions were visualized in the form of 0.5° resolution raster images for different APs. The images were then resampled to have the same spatial extent and origin as that of the (i) 100 m resolution LULC map and (ii) 30 m resolution SRTM-DEM to extract information on SVI_ME_ for the corresponding pixels in each continent. Variation in SVI_ME_ estimates corresponding to pixels of each LULC class was visualized in the form of a boxplot for each specified condition, intensity level and AP. Overall, 330 ($= 3 \times 2 \times 5 \times 11$) boxplots were prepared corresponding to each of the six continents. They correspond to the 3 intensity levels (moderate, severe, extreme), 2 conditions (dry/wet), 5 APs (i.e., 1, 3, 6, 12, and 24 months), and 11 LULC classes. The LULC classes include: (i) closed forest (L1) (ii) open forest (L2) (iii) shrublands (L3) (iv) herbaceous vegetation (L4) (v) herbaceous wetlands (L5) (vi) moss and lichen (L6) (vii) bare vegetation (L7) (viii) cropland (L8) (ix) built-up land (L9) (x) snow and ice (L10), and (xi) water bodies (L11). Medians of the boxplots corresponding to different LULC classes were considered for comparison of variability of wet and dry conditions (for various intensity levels and APs) across continents.

To analyze change in monthly and seasonal scale SVI_ME_ with elevation in each continent, a two-dimensional plot was prepared with the elevation of DEM pixels as abscissa versus the relative frequencies of their belongingness to different SVI_ME_ ranges as ordinate. Herein, the relative frequency corresponding to an elevation refers to the percentage of the total pixels in the continent having that elevation, and it is classified into five SVI_ME_ ranges (0–0.2, 0.2–0.4, 0.4–0.6, 0.6–0.8, and 0.8–1.0).

## Results and discussion

### Assessment of seasonal and monthly scale inter-annual variability

**Figure 1:**
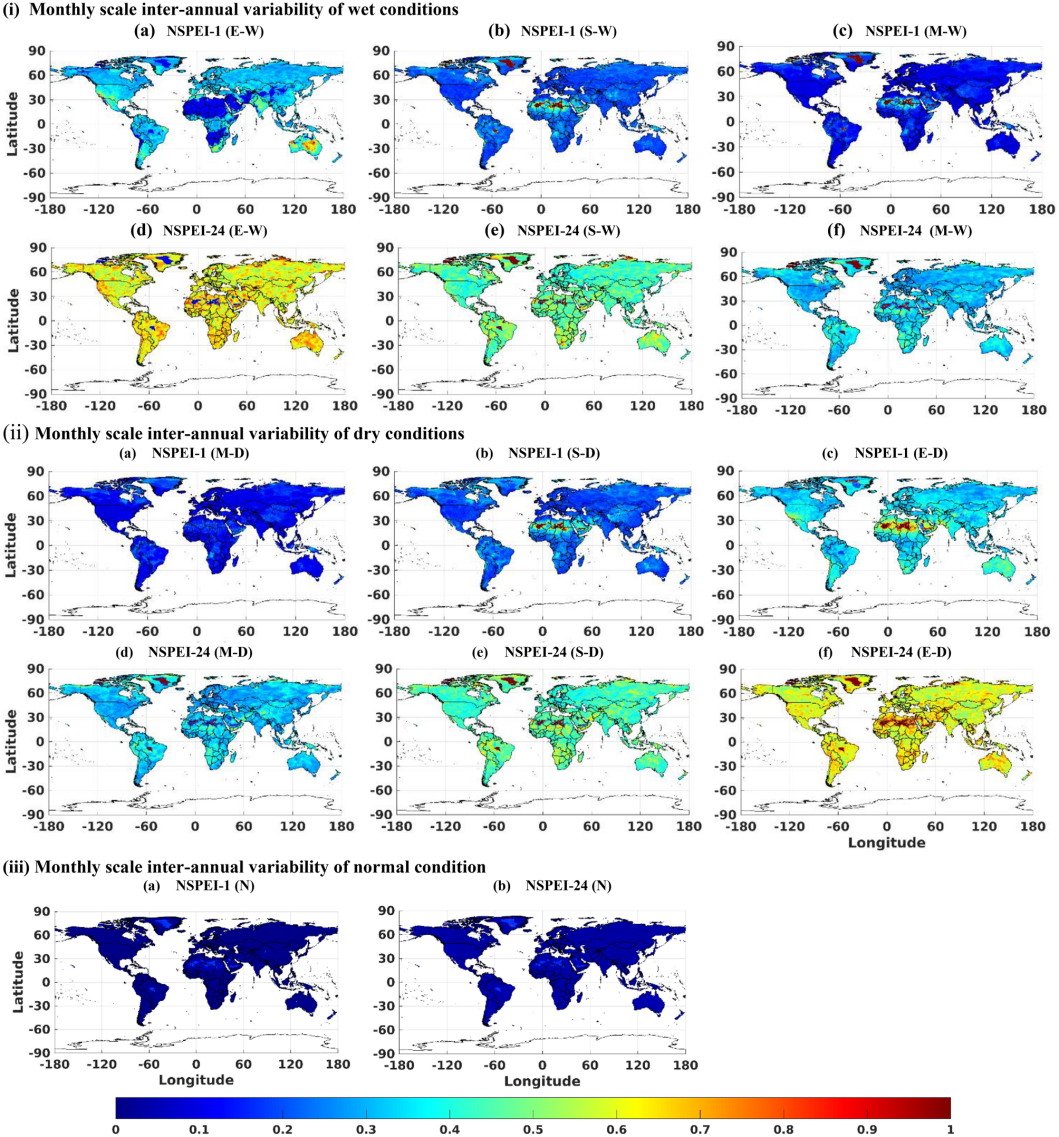
Monthly scale inter-annual variability (SVI_ME_) of (i) wet (W), (ii) dry (D), and (iii) normal (N) conditions for the period 1901 to 2019. In the notation NSPEI-*M*, *M* indicates the accumulation period (AP) in months. Extreme, severe and moderate-intensity levels of wet conditions are denoted by E-W, S-W, M-W, respectively. The corresponding intensity levels in the case of dry conditions are denoted by E-D, S-D, M-D, respectively. *The maps were generated using MATLAB (version: R2021a; https://www.mathworks.com/products/matlab.html ).

**Figure 2:**
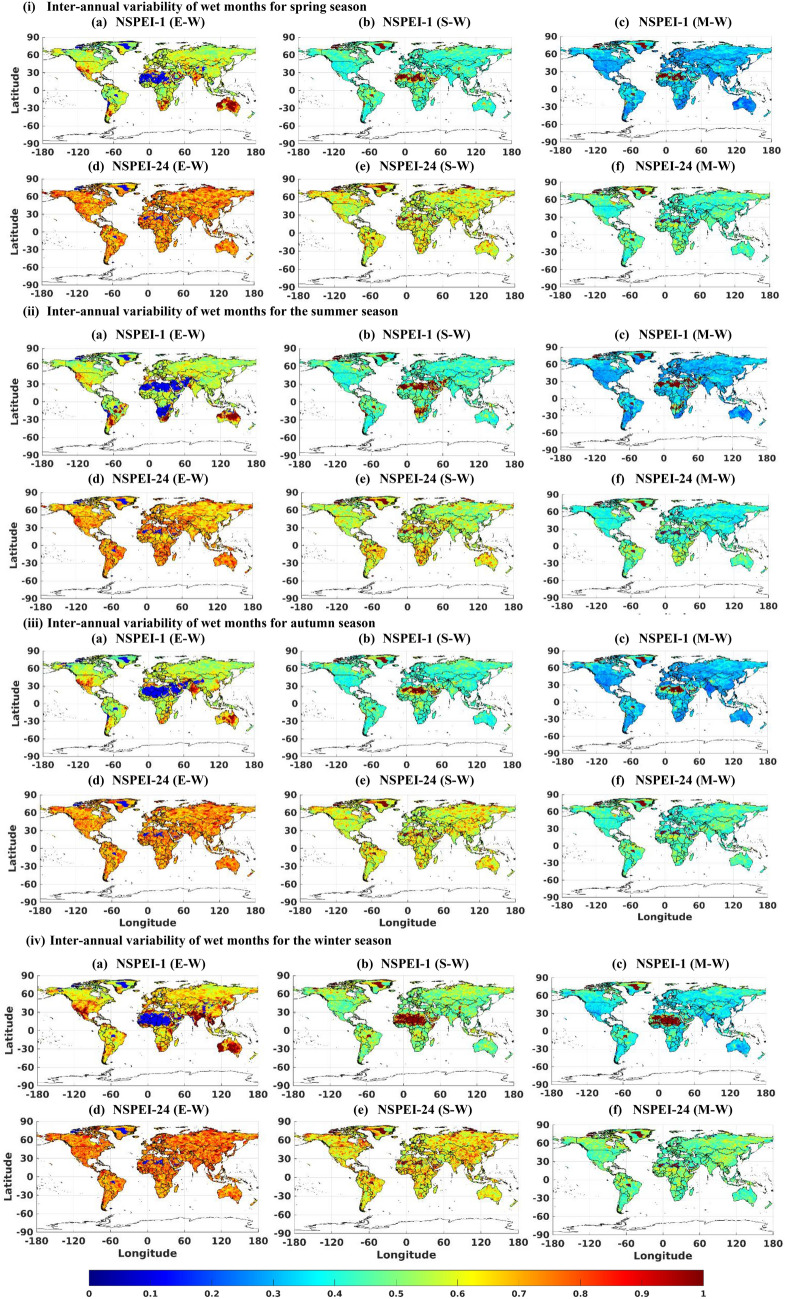
Inter-annual variability (SVI_ME_) of the variability of dry conditions in different seasons ((i) spring, (ii) summer, (iii) autumn and (iv) winter) during the period 1901 to 2019. In the notation NSPEI-*M*, *M* indicates the accumulation period (AP) in months. Extreme, severe and moderate-intensity levels of dry conditions are denoted by E-D, S-D, M-D, respectively. *The maps were generated using MATLAB (version: R2021a).

**Figure 3:**
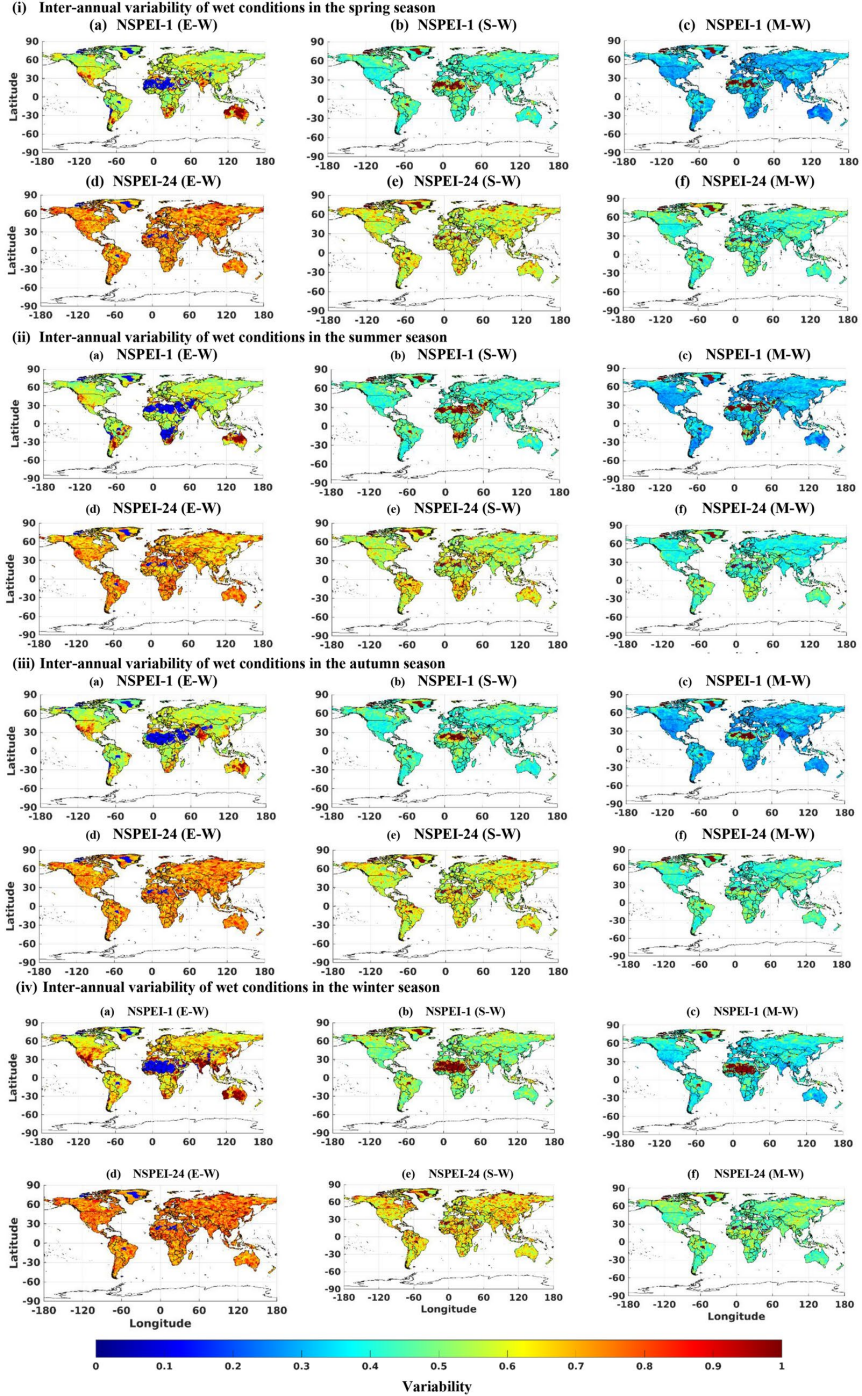
Inter-annual variability (SVI_ME_) of wet conditions in different seasons ((i) spring, (ii) summer, (iii) autumn and (iv) winter) during the period 1901 to 2019. In the notation NSPEI-*M*, *M* indicates the accumulation period (AP) in months. Extreme, severe and moderate-intensity levels of wet conditions are denoted by E-W, S-W, M-W, respectively. *The maps were generated using MATLAB (version: R2021a).

The monthly (Figs. 1, S3) and seasonal (Figs. 2, 3, S4, S5) scale variability (SVI_ME_) in the occurrence of wet and dry conditions of various intensity levels (moderate, severe and extreme) generally increase with APs for all the continents. This is mainly because long-duration drought/flood events (identified with a larger AP such as 12-, 24-month) lack uniformity/regularity in occurrence than short duration (e.g., 1-, 3-month) events. Furthermore, the magnitude of variability (SVI_ME_) generally increases with the intensity level (moderate, severe and extreme) of wet/dry conditions. This is expected, as extreme drought conditions that occur rarely are unlikely to be witnessed at uniform/regular intervals than relatively frequent moderate intensity drought conditions. In the case of normal conditions, SVI_ME_ was approximately zero for all the APs at both monthly and seasonal scales, indicating that the variability in the occurrence of normal months/seasons is nearly negligible for each AP (Figs. 1(iii), S6). It implies that almost all the continents have a nearly equal probability of experiencing the normal condition in all months/seasons. Furthermore, across all the APs, the highest variability (SVI_ME_) in the occurrence of both wet and dry conditions was in the winter season. This could be attributed to large anomalies in rainfall patterns (either severe droughts or heavy rainfall) due to large scale atmospheric circulations in different geographic regions of the globe during the winter season^47^.

There are few locations on the globe where the variability of SVI_ME_ deviates from the aforementioned general behaviour. Arid deserted regions (e.g., Sahara, Kalahari, Namib, Arabia, Turkestan, Gobi, Peruvian, and Arctic) experience low variability (i.e., SVI_ME_ tends to zero) in extreme wet conditions than moderate and severe wet conditions at both seasonal and monthly timescales, as these regions are unlikely to receive high precipitation in any of the months/seasons. Australia experiences higher variability (SVI_ME_) in extreme wet conditions over the one-month AP (i.e., smaller duration) than larger APs (i.e., longer durations) in all seasons. This could be attributed to high variability in the occurrence of short-duration extreme precipitation over this region (unlike other continents) due to the effect of different climate circulation patterns^48–52^ (such as the El Niño–Southern Oscillation (ENSO), Indian Ocean Dipole (IOD), and the southern annular mode (SAM)). Similarly, India experiences high variability in extreme wet conditions over the 1-month AP (see NSPEI-1) than larger APs during winter (highest) and autumn seasons (Figs. 3(iii) and 3(iv)a). High variability in the intensity of short duration rainfall is possibly due to the (i) western disturbances during the autumn and winter seasons in north-western parts (Western Himalayas and adjoining northern plains) of India, (ii) depressions and cyclonic storms that typically form in the Bay of Bengal and move north-westwards into the monsoon trough region over central India mainly during the early autumn season, and (iii) north-east trade winds in south-eastern parts of India that are predominant during the winter season^53–56^. The results indicate that the variability of short-term extreme wet conditions is increasing in central India, which substantiates the findings of Singh et al. (2014)^57^ that the frequency of extreme wet spells is decreasing, whereas their intensity is increasing over this region. The southern tip of Africa also experiences analogous high variability in extreme wet conditions over a 1-month AP (see NSPEI-1), during the NH's spring and summer seasons, with summer witnessing the highest variability (Figs. 3(i)a and 3(ii)a). This is possibly due to the occasional influence of ENSO with La Niña, which enhances wet conditions in this region, especially during the African summer season, which lasts from December to March^58–60^ (e.g., extreme summer rainfall during 1974 and 1976). The high variability of extreme wet conditions for 1-month AP in the southern parts of South America (during NH summer and spring) (Fig. 3(i)a and (ii)a) and western parts of the United States (during NH autumn and winter) (Fig3(iii)a and (iv)a) could also be due to the influence of ENSO^61,62^. Even though intense rainfall due to the influence of ENSO and other trade winds is likely to occur in other parts of the world, too, short-term variability of extreme wetness is high only in some regions. This is because these regions fall under arid/deserted zones, which otherwise receive very little rainfall over the entire year. Hence, the influence of these atmospheric circulation patterns can considerably influence the short-term variability of extreme/severe wet conditions on a seasonal scale.

**Figure 4:**
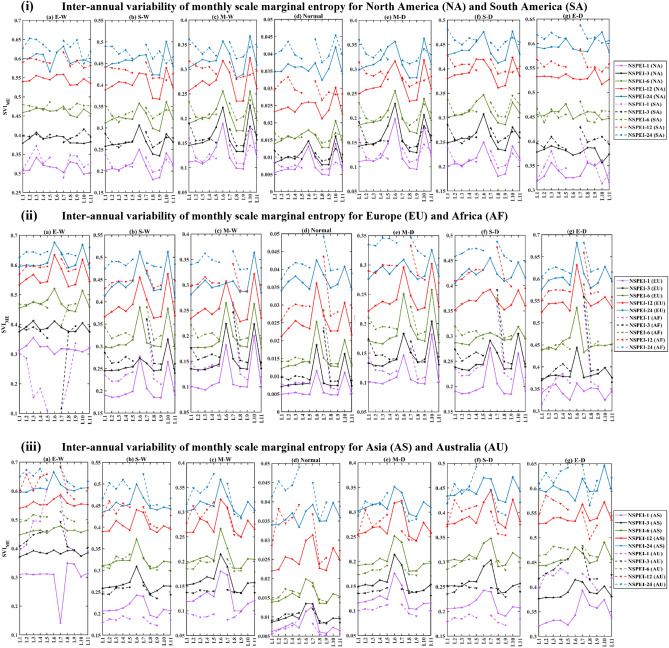
Monthly scale inter-annual variability (SVI_ME_) of wet, normal and dry conditions for different LULC classes in (i) North America and South America, (ii) Africa and Europe (iii) Asia and Australia. The LULC classes are L1: Closed Forest; L2: Open Forest; L3: Shrublands; L4: Herbaceous vegetation; L5: Herbaceous wetlands; L6: Moss and lichen; L7: Bare vegetation; L8: Cropland; L9: Built-up land; L10: Snow and ice; L11: Water bodies. In the notation NSPEI-*M*, *M* indicates the accumulation period (AP) in months. Extreme, severe and moderate-intensity levels of wet conditions are denoted by E-W, S-W, M-W, respectively. The corresponding intensity levels in the case of dry conditions are denoted by E-D, S-D, M-D, respectively. *The plots were generated using MATLAB (version: R2021a).

The variability of wet and dry conditions was also analyzed with respect to changes in location indicators (latitude, longitude, elevation) and LULC classes in each of the continents (except Antarctica). The percentage of the area falling in different LULC classes for each continent is provided in Table S3a. Analysis at monthly scale indicated that in North America, variability (SVI_ME_) in moderate and severe wet/dry conditions is generally highest for L6 (Moss and lichen) and L10 (Snow and ice) LULC classes for any chosen AP (Fig. 4i). In general, differences in SVI_ME_ are marginal across all other LULC classes for each AP. Variability (SVI_ME_) in the extreme wet condition is least for the L5 (Herbaceous wetlands) class over a 24-months AP (NSPEI-24 in Fig. 4i). In South America, variability (SVI_ME_) in intensity levels of wet and dry conditions has marginal differences across different LULC classes (Fig. 4i). However, short-duration extreme wet and dry conditions discernible for 1-month AP exhibit higher variability for L3, L8 and L9 classes (i.e., shrublands, cropland, built-up land) and lower variability for L1, L4, L7 and L10 classes (i.e., closed forest, herbaceous vegetation, bare vegetation, snow and ice). Across all the continents, variability (SVI_ME_) in the occurrence of wet and dry conditions generally decreases from extreme to moderate intensity levels. An exception is noted for the variability of extreme wet conditions corresponding to the 1-month AP in Africa (NSPEI-1 in Fig. 4(ii)a), which is found to be much lower compared to that of higher APs. The SVI_ME_ (variability) is generally highest for L7 class (Bare vegetation) in Africa, except for the extreme wet condition. In Europe, the highest variability (SVI_ME_) across all intensity levels corresponding to wet and dry conditions was evident for L6 (Moss and lichen) and L10 (Snow and ice) LULC classes (Fig. 4ii), analogous to North America. This increase in variability of L6 and L10 was found to be more prominent during moderate and severe wet/dry conditions across all APs. In Asia, slightly higher variability was evident in the case of (i) L6 (Moss and lichen) class for severe wet conditions, (ii) L6 and L7 (Bare vegetation) classes for moderate wet and dry conditions, (iii) L7 and L10 (Snow and ice) classes for extreme dry condition. In Asia, class L7 exhibited exceptionally lower variability (SVI_ME_) in the occurrence of extreme wet conditions over a one-month AP (NSPEI-1 in Fig. 4(iii)a). In Australia, no specific trend was evident in variability (SVI_ME_) of extreme wet condition for most of the LULC classes over different APs (Fig. 4iii). In general, over all the continents, variability in short and long duration severe and moderate wet/dry conditions is relatively higher for L6 (Moss and lichen) and L10 (Snow and ice) classes. This is evident in the analysis for most of the APs.

Analysis of variability (SVI_ME_) at seasonal scale revealed that in North America, variability (SVI_ME_) in short duration extreme wet and dry conditions is the highest for L3 (shrublands), L8 (cropland), and L9 (built-up land) classes in the NH winter season (NSPEI-1 in Figs. S7(i)a, S8(i)a). Furthermore, analysis of all the APs indicated that L6 (Moss and lichen) and L10 (Snow and ice) classes witness higher variability in extreme wet and dry conditions and lower variability in moderate and severe wet/dry conditions during all the seasons (Figs. S9(i), S10(i)). In general, over all the continents, the magnitude of the seasonal scale SVI_ME_ is highest for extreme intensity level and least for moderate intensity level in both wet and dry conditions (analogous to monthly scale SVI_ME_), irrespective of the chosen LULC class and AP, as expected. The L6 (Moss and lichen) and L10 (Snow and ice) LULC classes in Europe (Fig. S7(iv)) and L6 LULC class in Asia (Fig. S7(v)) show an exception to this general behaviour for the wet condition in various seasons. In Europe, the magnitude of SVI_ME_ is higher for L6 class (implying it experienced higher variability), corresponding to extreme dry conditions over a lower AP in NH winter and spring (NSPEI-1 and NSPEI-3 in Fig. S8(iv)a-b) and for higher AP (NSPEI-24 in Fig. S8(iv)e) in all seasons. In Asia, the L6 class exhibited higher (lower) variability for moderate (extreme) dry conditions discerned from higher APs (i.e., NSPEI-12 and NSPEI-24) in different seasons. Whereas for wet conditions of various intensity levels, higher variability (SVI_ME_) was found for the L6 class during all seasons. In general, globally, the LULC classes L5 (Herbaceous wetlands), L6 (Moss and lichen), L7 (bare vegetation) and L10 (snow and ice) are the most susceptible to variability in wet and dry conditions of different intensity levels over all the APs, and among those LULC classes, L6 and L10 showed the highest variability.

**Figure 5:**
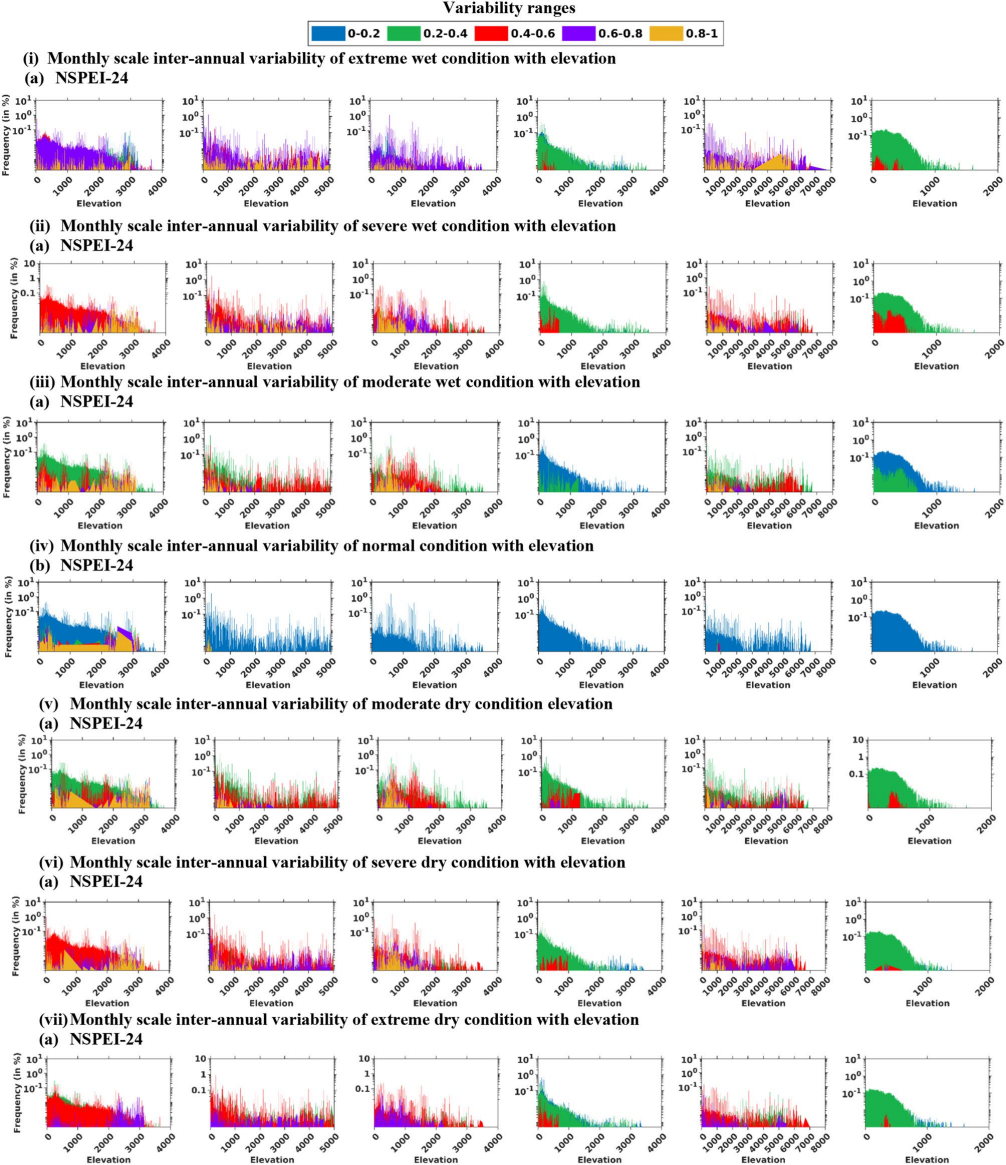
Monthly scale inter-annual variability (SVI_AE_) in wet conditions (i, ii, iii), normal condition (iv) and dry conditions (v, vi) for different elevation ranges in various continents. In the notation NSPEI-*M*, *M* indicates the accumulation period (AP) in months. *The plots were generated using MATLAB (version: R2021a).

Among the different elevation ranges, the highest percentage of the area in all the six continents falls in the elevation range 0–500 m. The percentage of the total area falling in each elevation range corresponding to different continents is provided in Table S3b. The analysis of the change in monthly and seasonal scale variability (SVI_ME_) with elevation showed that for North America, Europe, and Australia, the variability in dry/wet conditions decreases with an increase in elevation, and maximum variability occurs for lower elevation ranges (mainly 0–1000 m), whereas for South America, Africa and Asia no specific pattern is evident with change in the elevation (Figs. 5, S9). For Asia, there is an aberrant increase in the variability (SVI_ME_) for the elevation range of ~ 2000 to 5000 m (compared to other elevation ranges) for different APs. Thus, it could be considered a vulnerable elevation range for several intensity levels corresponding to wet/dry conditions. This can be noted from NSPEI-1 (Fig. 5(i)a), NSPEI-3 and NSPEI-24 (Fig. 5(i)b) for monthly extreme wet conditions, NSPEI-3 for monthly severe wet conditions, NSPEI-3 and -6 for the severe wet condition in summer.

**Figure 6:**
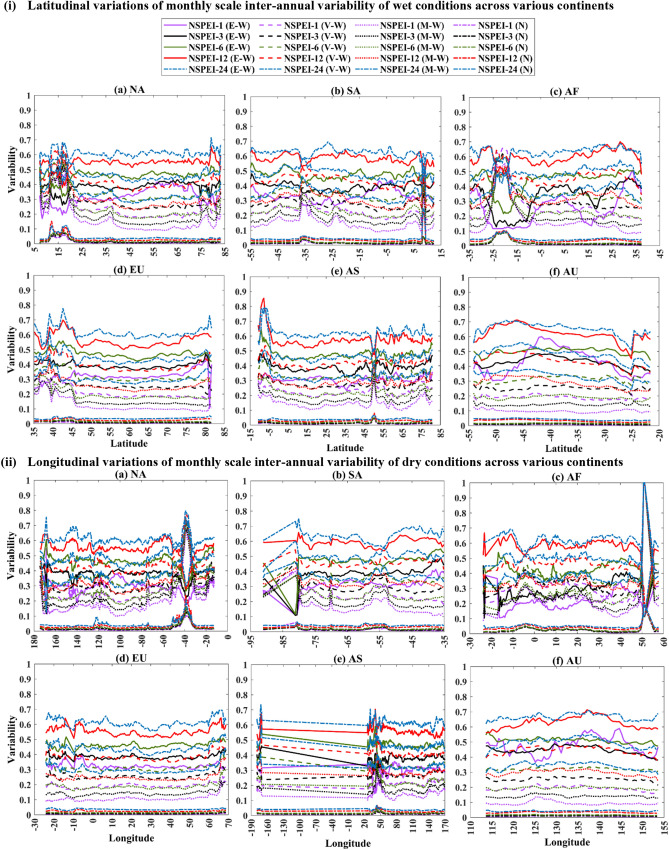
(i) Latitudinal and (ii) Longitudinal variation of monthly scale inter-annual variability (SVI_ME_) of wet conditions across North America (NA), South America (SA), Europe (EU), Africa (AF), Asia (AS) and Australia (AU). *The plots were generated using MATLAB (version: R2021a).

The analysis of the variability of SVI_ME_ with latitude and longitude (Fig. 6) showed that there is a considerable irregularity in variability (SVI_ME_) of wet conditions at monthly scale in various latitudinal ranges, including (i) ~ 10–20°N and ~ 75–83°N for North America, (ii) 34°–30°S and 8.5°–9.5°N for South America, (iii) 28°–5°S for Africa, (iv) 35°–46°N and 80.5°-81.5°N for Europe, (v) 10°–10.7°S and 45°–53°N for Asia. Similarly, longitudinal ranges exhibiting considerable irregularity include (i) 174°–163°W and 45°-30°W for North America, (ii) 91°–78°W for South America, (iii) 23.5°–15.7°W and 49°- 56°E for Africa and (iv) 179.5°W to 170°E and 27°–66°E for Asia. The variability of the dry condition has a considerable irregularity over the latitudinal ranges (i) 11°–21°N and 74°–83°N in North America, (ii) 7°–12.4°N in South America, (iii) 28°-5°S in Africa, (iv) 35°–46°N in Europe and (v) 10.7°–1°S and 45°-53°N in Asia. Similarly, the variability is notable over the longitude ranges (i) 174°–167°W and 60°-12.5°W in North America, (ii) 91°–78°W in South America, (iii) 49°–56°E in Africa and (iv) − 179.5°S to 170°N and 27°–66°E for Asia. The seasonal scale variability also exhibited nearly the similar behaviour as monthly scale variability for all the continents. However, there were no substantial irregularities in monthly and seasonal scale SVI_ME_ estimates for wet and dry conditions along the longitude in Europe and latitude and longitude in Australia. This is possibly because, in Europe, the sub-regional climate characteristics are mainly dependent on latitudinal variations^63^ due to the influence of large-scale atmospheric circulations (e.g., North Atlantic Oscillation^64^, Scandinavian pattern^65^ and atmospheric blocking^66,67^). Whereas in the case of Australia, which is the smallest continent surrounded by the Indian Ocean, the latitudinal and longitudinal variability of SVI_ME_ is expected to be less relative to other continents, as different parts of the continent are equally susceptible to different climatic variations.

### Assessment of intra-annual variability

**Figure 7:**
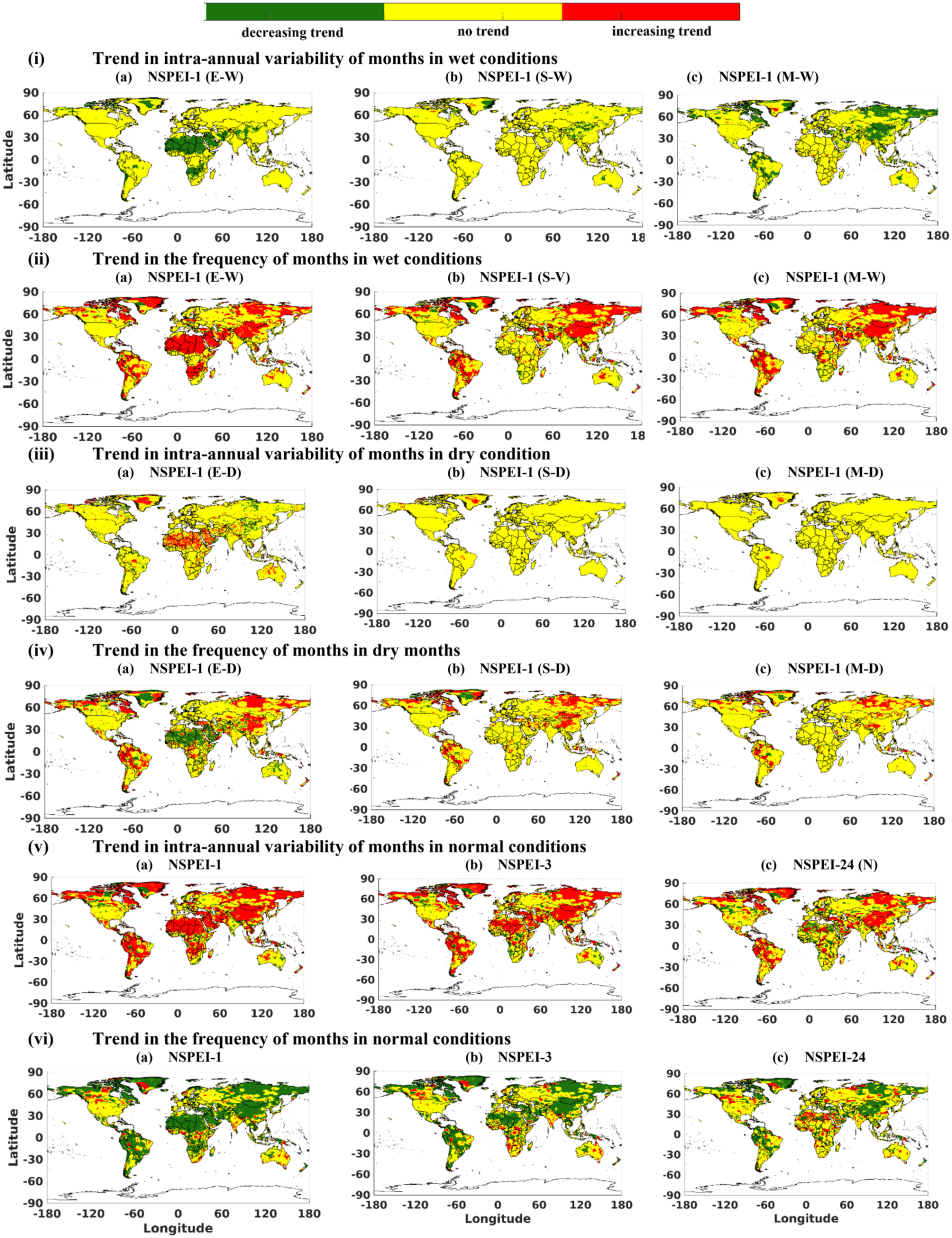
Trends in intra-annual variability (SVI_AE_) and frequency/count of months in wet conditions (i, ii), dry conditions (iii, iv) and normal condition (iv) identified corresponding to different accumulation periods (APs) in analysis with Mann–Kendall (MK) trend test considering 5% significance level. *The maps were generated using MATLAB (version: R2021a).

The estimates of SVI_AE_ and frequency of months in each year exhibiting normal and wet/dry conditions of various intensity levels were determined for 0.5° resolution CRU grids for the baseline period (1901–2019) by considering different APs. Furthermore, trends of SVI_AE_ and frequency/count of months corresponding to each condition (wet/normal/dry) were determined using the MK trend test considering a 5% significance level (Fig. 7, S10). A decreasing trend in variability (SVI_AE_) for wet conditions and a contrasting increasing trend in the same for the dry conditions was evident for most grids in all continents for different intensity levels and APs. In contrast, the frequency/count of months in wet conditions showed an increasing trend, and the same for months in dry conditions showed a decreasing trend. In the case of months in normal condition, an increasing trend in variability (SVI_AE_) and decreasing trend in frequency was observed. In general, globally, the trends of SVI_AE_ and frequency follow an inverse relationship, i.e., when variability (SVI_AE_) of wet/normal/dry conditions increases, its frequency decreases at the corresponding location/grid. However, the converse is not always found to be true, as there are many grids exhibiting trends in frequency but not in variability. At those grids, one could expect an increasing/decreasing trend of variability (SVI_AE_) in future based on the existing regional pattern of the trend in the frequency of months with wet/normal/dry conditions. In general, the locations/grids exhibiting trends in both variability (SVI_AE_) and frequency of months having dry and/or wet conditions are decreasing with an increase in the AP (Figs. S10 i-iv). However, the number of grids exhibiting a negative (positive) trend in variability (SVI_AE_) corresponding to the normal condition is increasing (decreasing) with an increase in the AP (Fig. S10v). Whereas the grids having the positive (negative) trend in the frequency of months having normal conditions are increasing (decreasing) with an increase in AP (Figs. S10v, vi). However, there is an exception from the aforementioned general behaviour for dry conditions in the arid regions of the Sahara Desert, Africa. The regions have more grids exhibiting trends in frequency and variability (SVI_AE_) of months in an extremely dry condition for 24 months AP than for 12- and 6-months APs (see NSPEI-6, -12, -24 months in Figs. S10iii, iv).

Global analysis of various intensity levels of wet conditions corresponding to different APs indicated that the number of grids exhibiting trends in variability and frequency was more for moderate-intensity levels and relatively less for severe and extreme intensity levels (in that order), except for 1-month AP (NSPEI-1) (Fig. 7i, ii). The anomalous behaviour for NSPEI-1 could be possibly due to the increasing trends of frequency and variability (SVI_AE_) for months in extreme wet conditions over Africa, especially in deserted regions of Sahara, Namib and Kalahari for the lower AP. Analogous global analysis on intensity levels of dry conditions showed contrasting observations. The number of grids exhibiting trends in variability and frequency was highest for extreme dry intensity levels and relatively less for severe and moderate dry intensity levels, in that order (Fig. 7iii, iv).

### Assessment of variability at decadal scale

**Figure 8:**
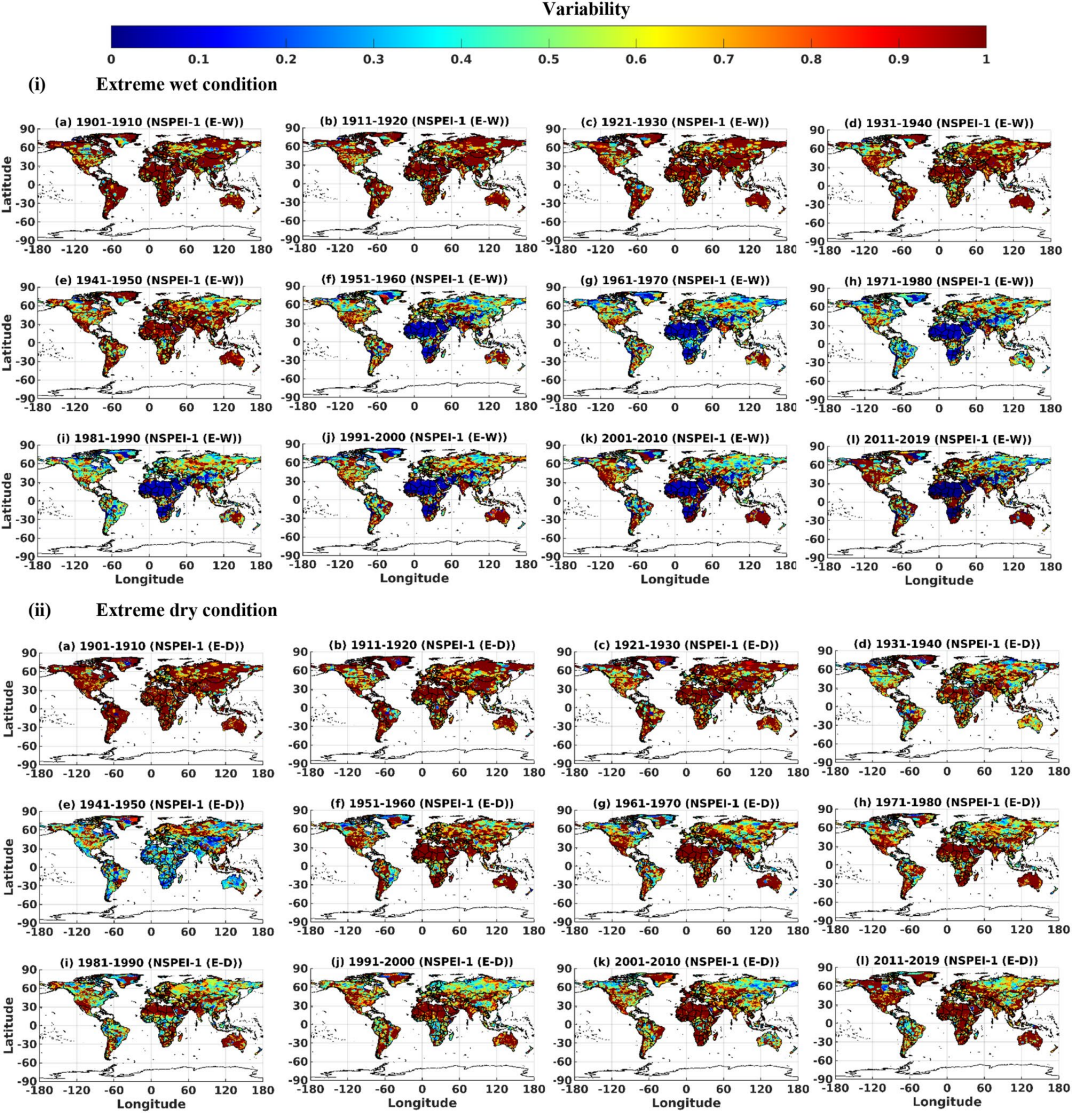
Decadal scale variability (SVI_DE_) of extreme (i) wet (E-W) and dry (E-D) conditions for 1-month accumulation period (AP). *The maps were generated using MATLAB (version: R2021a).

Decadal scale variability in normal conditions and various intensity levels corresponding to wet/dry conditions was determined in terms of SVI_DE_ for the 12 decades during 1901–2019 by considering different APs (Figs. 8 and S11-S16). The first 5 decades during 1901–1950 showed marginal differences in variability (SVI_DE_) of wet conditions (moderate, severe, extreme intensity levels) for all the APs. The highest and lowest values of SVI_DE_ were obtained for the first and the fifth decade, respectively. Thereafter the variability decreased gradually till the 1980s, and this decrease was more prominent for lower APs. The decrease could be due to an increase in the frequency/count of heavy precipitation events, especially in higher latitudes and tropical regions and during winters in the northern mid-latitudes, particularly since the 1950s^68,69^. Moreover, an increase in precipitation events can reduce negative soil moisture anomalies and minimize the risk of heatwave flash drought^70^, reducing variability in wet conditions. Analysis of dry conditions revealed that the first three decades (i.e., 1901–1930) showed marginal differences in variability (SVI_DE_). The variability was maximum for the first decade and minimum for the third decade, which gradually decreased thereafter till 1950. This decrease in drought variability could be attributed to more frequent drought events in the early 1930s, which resulted in the intensification of evapotranspiration driven by higher temperatures. This is very common, especially for lower APs (e.g., formation of heatwave flash drought) and humid and semi-humid regions. For example, the increased Arctic warming in the 1920s to 1940s which was mostly concentrated in higher latitudes^71^ (near-polar regions), the cold European winters^72^ in 1940–1942, the "Dust Bowl droughts and extreme heatwaves in North America^73–76^ in 1930s, the World War II period droughts in Australia^77^ between 1937 and 1945 and European summer droughts and heatwaves^78^ in 1940s. The changes in the wet and dry conditions in the latter decades could be attributed to global warming and the associated climate change.

Among all the decades from 1901-to 2019, those during 1951–1980 showed the lowest variability (SVI_DE_) of extreme, severe and moderate wet conditions. However, the variability gradually increased in the decades following 1990. A similar analysis performed on extreme, severe, and moderate dry conditions revealed the lowest variability for the decades during 1931–1950, and the highest was during the periods 1901–1910 and 1951–1980. In the recent decade (i.e., 2011–2019), both wet and dry conditions showed an increase in variability compared to the preceding decade (i.e., 2001–2010). Moreover, as expected, both wet and dry conditions showed an increase in variability with the increase in APs (highest for 24-month and lowest for 1-month) and intensity levels (highest for extreme and lowest for moderate conditions) for all the decades during 1901–2019.

## Summary and conclusions

Global-scale variability of different intensity levels (moderate, severe, extreme) of dry and wet conditions and normal conditions was analyzed by performing entropy-based analysis on estimates of NSPEI corresponding to different APs (1, 3, 6, 12, and 24 months) and intensity levels (moderate, severe, extreme). In general, at both monthly and seasonal scales, with an increase in AP and intensity level, the variability (SVI_ME_) is (i) increasing for dry and wet conditions, (ii) marginal for the normal condition, (iii) highest in the NH winter season (December to February). However, there exist few anomalies in this general behaviour. Variability in extreme wet conditions is least over the arid desert regions of the globe at both seasonal and monthly time-scales. It is highest over one-month AP in (i) Australia during all seasons, (ii) India during NH's winter (highest) and autumn seasons, (iii) southern tip of Africa during NH's spring and summer seasons, (iv) South America during the NH's summer and spring and (v) western parts of United States during NH's autumn and winter. These anomalies in the variability of short duration wet conditions could be mainly attributed to variations in the climate change-induced atmospheric circulation patterns.

Analysis of the variability (SVI_ME_) associated with various LULC classes showed that, in general, globally, the L5 (Herbaceous wetlands), L6 (Moss and lichen), L7 (bare vegetation), and L10 (snow and ice) classes are the most susceptible to variability in wet and dry conditions of different intensity levels over all the APs, and among those classes, L6 and L10 showed the highest variability. It is to be mentioned that the inferences drawn in this study are conditional on the uncertainties associated with LULC classification of CGLS-LC discrete global LC map. The analysis on changes in variability with elevation showed that, among different continents, North America, Europe, and Australia show a decrease in variability (SVI_ME_) of dry/wet conditions with an increase in elevation, and maximum variability occurs for lower elevation ranges (mainly 0–1000 m). However, for South America, Africa and Asia, no specific pattern is evident with change in the elevation. The analysis on the variability of SVI_ME_ with latitude and longitude showed a considerable irregularity in variability (SVI_ME_) of wet conditions at monthly scale in various latitudinal and longitudinal ranges for different continents. However, there were no substantial irregularities in monthly and seasonal scale SVI_ME_ estimates for wet and dry conditions with change in longitude for Europe and change in latitude and longitude for Australia.

Trend analysis on the frequency/count of dry and wet conditions indicated that wet and dry months are increasing worldwide. However, the frequency of short-term dry spells (identified with lower APs) is decreasing in arid/snowy regions (e.g., Sahara Desert, Greenland). Annual scale variability (SVI_AE_) of (i) wet conditions showed a decreasing trend, (ii) dry conditions exhibited an increasing trend. A majority of the locations showed a decreasing trend in the frequency and an increasing trend in variability for normal conditions. This clearly indicates that climate change is affecting the frequency and variability of extreme events (i.e., dry/wet spells).

Analysis of the decadal-scale variability of wet/normal/dry conditions indicated a noticeable decrease in variability of wet conditions from the 1950s and dry conditions from the 1930s. This is attributable to the fact that changes due to climate and global warming started to have a pronounced impact on wet and dry conditions in various parts of the globe from the 1950s and 1930s, respectively. In recent two decades, there has been a marginal increase in the variability of wet and dry conditions. However, it is less than what was evident prior to the 1950s/1930s on the global scale.

This study could be extended to analyze the changes in variability of dry, normal and wet conditions at global, continental, and regional scales considering (i) various future climate change scenarios (ii) flexibility in the time window of each season across different geographical locations (iii) finer time scales (e.g., daily) by modifying the entropy indices based on the practical implications and (iv) other types of droughts (e.g., hydrological). This could help in identifying various regions that are highly vulnerable to changes in climate and aid in planning effective mitigation measures.

## Supplementary Information

Supplementary Information.
