# Supplementary material for: Global assessment of spatiotemporal variability of wet, normal and dry conditions using multiscale entropy-based approach
Source: Sci Rep. 2022 Jun 13;12:9767. doi: 10.1038/s41598-022-13830-w (PMC9192772; doi:10.1038/s41598-022-13830-w)
Supplement: Supplementary file 1 — Supplementary Information. [file 41598_2022_13830_MOESM1_ESM.docx]

**Global Assessment of Spatiotemporal Variability of Wet, Normal and Dry Conditions using Multiscale Entropy-based Approach**

**Vijay Sreeparvathy^1*^, V. V. Srinivas^1,2,3^**

*^1^ Department of Civil Engineering, Indian Institute of Science, Bangalore 560 012, India*

*^2^ Interdisciplinary Centre for Water Research* (*ICWaR*)*, Indian Institute of Science,*

*^3^ Divecha Centre for Climate Change, Indian Institute of Science,*

*Bangalore 560 012, India*

*^*^*Corresponding author: Sreeparvathy Vijay (sreeparvathy[@iisc.ac.in](mailto:vvs@iisc.ac.in))

**Supplementary document**

**Contents of this file**

**Section S1:** Synthetic examples detailing the methodology and physical interpretation of variability of normal/wet/dry conditions

**Table S1.** Computation of variability corresponding to ED, SD, MD, normal, MW and SW conditions using marginal entropy (equation 2 in the main manuscript) and SVI (equation 5 in the main manuscript) for the considered 6 typical years (1990-1995). The maximum entropy ( ) is estimated to be equal to 2.58.

**Table S2:** Classification of dry/wet condition based on Non-stationary Standardized Precipitation Evapotranspiration Index (NSPEI).

**Table S3**: Percentage of the area falling in different (a) LULC classes and (b) elevation ranges for various continents.

**Figure S1.** The time series of (i)$B_{\text{ED, L1}}^{NSPEI}$, $B_{\text{SD, L2}}^{NSPEI}$, (ii) $B_{\text{MD, L3}}^{NSPEI}$, $B_{\text{normal, L4}}^{NSPEI}$and (iii) $B_{\text{MW, L5}}^{NSPEI}$ $B_{\text{SW, L6}}^{NSPEI}$ The subplots (iv), (v) and (vi) show the annual probability of occurrence ($\frac{m_{\upsilon}}{S}$) of specified events at a monthly timescale at locations L1 to L6.

**Figure S2.** Flowchart depicting the methodology and data used in the study

**Figure S3**. Monthly scale inter-annual variability (SVI_ME_) of (i) wet (W), (ii) dry (D), and (iii) normal (N) conditions for the period 1901 to 2019. In the notation NSPEI-*M*, *M* indicates the accumulation period in months. Extreme, severe and moderate-intensity levels of wet conditions are denoted by E-W, S-W, M-W, respectively. The corresponding intensity levels in the case of dry conditions are denoted by E-D, S-D, M-D, respectively.

**Figure S4.** Inter-annual variability (SVI_ME_) of wet conditions for different seasons ((i) spring, (ii) summer, (iii) autumn and (iv) winter during the period 1901 to 2019. In the notation NSPEI-*M*, *M* indicates the accumulation period in months. Extreme, severe and moderate-intensity levels of wet conditions are denoted by E-W, S-W, M-W, respectively.

**Figure S5.** Inter-annual variability of the variability (SVI_ME_) of dry conditions for different seasons ((i) spring, (ii) summer, (iii) autumn and (iv) winter) during the period 1901 to 2019. In the notation NSPEI-*M*, *M* indicates the accumulation period in months. Extreme, severe and moderate-intensity levels of dry conditions are denoted by E-D, S-D, M-D, respectively.

**Figure S6:** Inter-annual variability (SVI_ME_) of normal condition for (i) spring (ii) summer, (iii) autumn and (iv) winter season for the period 1901-2019.

**Figure S7.** Variation of seasonal scale inter-annual variability (SVI_ME_) of wet condition for different LULC classes for (i) North America (ii) South America (iii) Africa (iv) Europe (v) Asia (vi) Australia.

**Figure S8.** Variation of seasonal scale inter-annual variability (SVI_ME_) of dry condition for different LULC classes for (i) North America (ii) South America (iii) Africa (iv) Europe (v) Asia (vi) Australia

**Figure S9.** Monthly scale inter-annual variability (SVIAE) in wet conditions (i, ii, iii), normal condition (iv) and dry conditions (v, vi) for different elevation ranges in various continents. In the notation NSPEI-M, M indicates the accumulation period (AP) in months.

**Figure S10.** Trends in intra-annual variability (SVI_AE_) and frequency/count of months in wet conditions (i, ii), dry conditions (iii, iv) and normal conditions (v, vi) identified corresponding to different accumulation periods in analysis with the Mann-Kendall (MK) trend test considering 5% significance level.

**Figure S11.** Decadal scale variability (SVI_DE_) of moderate wet (M-W) conditions across different accumulation periods.

**Figure S12.** Decadal scale variability (SVI_DE_) of severe wet (S-W) conditions across different accumulation periods.

**Figure S13.** Decadal scale variability (SVI_DE_) of extreme wet (E-W) conditions across different accumulation periods.

**Figure S14.** Decadal scale variability (SVI_DE_) of moderately dry (M-D) conditions across different accumulation periods.

**Figure S15.** Decadal scale variability (SVI_DE_) of severe dry (S-D) conditions across different accumulation periods.

**Figure S16.** Decadal scale variability (SVI_DE_) of extreme dry (E-D) conditions across different accumulation periods.

**Section S1: Synthetic examples of the methodology and physical interpretation of variability of normal/wet/dry conditions**

In this section, we have provided some synthetic examples to explain (a) the methodology considered for estimating the variability of dry/wet/normal conditions using entropy indices and (b) its physical interpretation. Let $Y_{i}^{\text{NSPEI}}=\left\{y_{1,i}^{\text{NSPEI}},\ldots,y_{t,i}^{\text{NSPEI}},\ldots y_{L,i}^{\text{NSPEI}}\right\}$ denote NSPEI timeseries corresponding at site *i* corresponding to one month accumulation period. The timeseries are transformed into a binary format $B_{^{c,i}}^{NSPEI}=\left\{b_{1,c,i}^{\text{NSPEI}},\ldots,b_{t,c,i}^{\text{NSPEI}},\ldots b_{L,c,i}^{\text{NSPEI}}\right\}$. The $b_{t,c,i}^{\text{NSPEI}}$, which corresponds to $y_{t,i}^{\text{NSPEI}}$, is assigned value 1 if the site *i* experiences a specified condition *c* (Table S1) in month *t*, and zero otherwise. The condition *c* could be extreme dry (ED), severe dry (SD), moderate dry (MD), normal, moderate wet (MW), severe wet (SW) or extreme wet (EW).

Suppose the study area comprises six locations (L1 to L6), each having timeseries corresponding to one month AP for 6 typical years (1990 to 1995). Identify ED, SD, MD, N, MW, SW conditions at L1 to L6, respectively, to arrive at binary timeseries ($B_{\text{ED, L1}}^{NSPEI}$, $B_{\text{SD, L2}}^{NSPEI}$, $B_{\text{MD, L3}}^{NSPEI}$, $B_{\text{N, L4}}^{NSPEI}$, $B_{\text{MW, L5}}^{NSPEI}$ and $B_{\text{SW, L6}}^{NSPEI}$). The annual occurrence of the specified condition at different locations is considered to follow one of the three scenarios.

- In scenario 1, the ED and SD conditions occur regularly in a specific month of a year, implying the probability of occurrence of each condition is uniformly distributed across years. Suppose ED condition occurs in April month at L1 and SD condition occurs in May month at L2 (Figure S1 i, iv).
- In scenario 2, the MD and normal conditions occur every year but in the random month(s). Here too, the probability of occurrence of each condition is uniformly distributed across years. Suppose MD condition occurs every year in a random month at L3, whereas the normal condition occurs at L4 in ten random months every year (Figure S1 ii, v).
- In scenario-3, the chosen conditions (MW and SW) occur at random across years with non-uniform recurrence intervals (or annual frequency/count). Suppose L5 and L6 experience these conditions (Figure S1 iii, vi).

**Table S1.** Computation of variability corresponding to ED, SD, MD, normal, MW and SW conditions using marginal entropy (equation 2 in the main manuscript) and SVI (equation 5 in the main manuscript) for the considered 6 typical years (1990-1995). The maximum entropy $H_{\max}$ ($=\log_{2}N$) is estimated to be equal to 2.58.

| Year | ED condition (L1) | | SD condition (L2) | | MD condition (L3) | | normal condition (L4) | | MW condition (L5) | | SW condition (L6) | |
| --- | --- | --- | --- | --- | --- | --- | --- | --- | --- | --- | --- | --- |
|  | $\frac{m_{\upsilon}}{S}$ | $\text{-}\frac{m_{\upsilon}}{S}\text{log}_{2}\left[\frac{m_{\upsilon}}{S}\right]$ | $\frac{m_{\upsilon}}{S}$ | $\text{-}\frac{m_{\upsilon}}{S}\text{log}_{2}\left[\frac{m_{\upsilon}}{S}\right]$ | $\frac{m_{\upsilon}}{S}$ | $\text{-}\frac{m_{\upsilon}}{S}\text{log}_{2}\left[\frac{m_{\upsilon}}{S}\right]$ | $\frac{m_{\upsilon}}{S}$ | $\text{-}\frac{m_{\upsilon}}{S}\text{log}_{2}\left[\frac{m_{\upsilon}}{S}\right]$ | $\frac{m_{\upsilon}}{S}$ | $\text{-}\frac{m_{\upsilon}}{S}\text{log}_{2}\left[\frac{m_{\upsilon}}{S}\right]$ | $\frac{m_{\upsilon}}{S}$ | $\text{-}\frac{m_{\upsilon}}{S}\text{log}_{2}\left[\frac{m_{\upsilon}}{S}\right]$ |
| 1990 | 1/6 | 0.43 | 1/6 | 0.43 | 1/6 | 0.43 | 10/60 | 0.43 | 3/17 | 0.44 | 2/24 | 0.30 |
| 1991 | 1/6 | 0.43 | 1/6 | 0.43 | 1/6 | 0.43 | 10/60 | 0.43 | 1/17 | 0.24 | 2/24 | 0.30 |
| 1992 | 1/6 | 0.43 | 1/6 | 0.43 | 1/6 | 0.43 | 10/60 | 0.43 | 2/17 | 0.36 | 1/24 | 0.19 |
| 1993 | 1/6 | 0.43 | 1/6 | 0.43 | 1/6 | 0.43 | 10/60 | 0.43 | 4/17 | 0.49 | 7/24 | 0.52 |
| 1994 | 1/6 | 0.43 | 1/6 | 0.43 | 1/6 | 0.43 | 10/60 | 0.43 | 5/17 | 0.52 | 8/24 | 0.53 |
| 1995 | 1/6 | 0.43 | 1/6 | 0.43 | 1/6 | 0.43 | 10/60 | 0.43 | 2/17 | 0.36 | 4/24 | 0.43 |
| Total (*H*^ME^) | - | 2.58 | - | 2.58 | - | 2.58 | - | 2.58 |  | 2.42 |  | 2.27 |
| SVI | - | 0 | - | 0 | - | 0 | - | 0 |  | 0.063 |  | 0.12 |

| (i) | (iv) |
| --- | --- |
|  |  |
| (ii) | (v) |
|  |  |
| (iii) | (vi) |
|  |  |

**Figure S1.** The time series of (i)$B_{\text{ED, L1}}^{NSPEI}$, $B_{\text{SD, L2}}^{NSPEI}$, (ii) $B_{\text{MD, L3}}^{NSPEI}$, $B_{\text{normal, L4}}^{NSPEI}$and (iii) $B_{\text{MW, L5}}^{NSPEI}$ $B_{\text{SW, L6}}^{NSPEI}$ The subplots (iv), (v) and (vi) show the annual probability of occurrence ($\frac{m_{\upsilon}}{S}$) of specified events at a monthly timescale at locations L1 to L6.

The results indicate that the inter-annual uncertainty at a monthly timescale (*H*^ME^) for a chosen condition is maximum (or variability is minimum) when the condition occurs (a) regularly in a specific month of each year (Scenario-1) or (b) irregularly in a random month every year (Scenario-2). In both scenarios, the probability of occurrence of the conditions follows a uniform distribution. Furthermore, the *H*^ME^ decreases when the probability of annual occurrence of the specified condition deviates from the uniform distribution (Scenario-3).

To gain further insight into the inter-annual variability of a condition in a given month, the month’s marginal entropy can be estimated as follows,

$$H_{^{\tau}}^{\text{ME}}=-\sum_{\upsilon=1}^{N} \frac{m_{\upsilon}^{\tau}}{S^{\tau}}\text{log}_{2}\left[\frac{m_{\upsilon}^{\tau}}{S^{\tau}}\right]\text{ , where }S^{\tau}=\sum_{\upsilon=1}^{N} m_{\upsilon}^{\tau}$$

where $m_{\upsilon}^{\tau}=1$ if the month $\tau$ in year $\upsilon$ has a specified condition (i.e., normal, moderate/severe/extreme wet or dry condition) and zero otherwise, *N* represents the total number of years. One can try computing the $H_{^{\tau}}^{\text{ME}}$ corresponding to different months at a global/regional/local scale to assess the uncertainty associated with each month. However, the results to be analysed on a global scale would be voluminous. Hence, this is deferred for future research. It is to be noted that the estimation of decadal entropy and apportionment entropy also follows the same concept as explained in the synthetic example.

**Table S2:** Classification of dry/wet conditions based on Non-stationary Standardized Precipitation Evapotranspiration Index (NSPEI).

| **NSPEI values** | **Climatic category** |
| --- | --- |
| NSPEI > 2 | Extreme wet |
| 1.5< NSPEI ≤2 | Severe wet |
| 1< NSPEI ≤1.5 | Moderate wet |
| -1< NSPEI ≤1 | Normal |
| -1.5< NSPEI ≤-1 | Moderate dry |
| -2< NSPEI ≤-1.5 | Severe dry |
| NSPEI ≤-2 | Extreme dry |

**Table S3**: Percentage of the area falling in different (a) LULC classes and (b) elevation ranges for various continents.

(a)

| Continents | LULC classes | | | | | | | | | | |
| --- | --- | --- | --- | --- | --- | --- | --- | --- | --- | --- | --- |
|  | L1 | L2 | L3 | L4 | L5 | L6 | L7 | L8 | L9 | L10 | L11 |
| North America | 25.29 | 8.05 | 8.53 | 20.53 | 1.30 | 7.53 | 1.96 | 7.69 | 0.51 | 13.60 | 5.00 |
| South America | 43.95 | 4.32 | 10.77 | 23.48 | 1.58 | 0.00 | 6.64 | 7.72 | 0.26 | 0.26 | 1.03 |
| Africa | 14.44 | 12.93 | 13.94 | 13.92 | 0.62 | 0.00 | 34.91 | 8.30 | 0.18 | 0.00 | 0.77 |
| Europe | 40.27 | 5.34 | 1.14 | 14.84 | 2.56 | 0.31 | 0.67 | 29.21 | 1.63 | 1.02 | 3.02 |
| Asia | 32.35 | 2.78 | 1.83 | 28.18 | 3.61 | 0.63 | 15.77 | 11.91 | 0.58 | 0.26 | 2.11 |
| Australia | 10.34 | 12.38 | 15.48 | 55.00 | 0.17 | 0.00 | 1.14 | 5.24 | 0.13 | 0.00 | 0.12 |

(b)

| Continents | Elevation ranges (m) | | | | | |
| --- | --- | --- | --- | --- | --- | --- |
|  | [0,100] | (100,500] | (500,1000] | (1000,2000] | (2000,5000] | (5000, ∞) |
| North America | 12.8 | 40.6 | 17.8 | 17.8 | 10.9 | 0.0 |
| South America | 21.3 | 49.3 | 16.0 | 5.8 | 7.5 | 0.1 |
| Africa | 5.8 | 42.0 | 30.0 | 21.2 | 1.1 | 0.0 |
| Europe | 28.3 | 57.5 | 10.3 | 3.3 | 0.6 | 0.0 |
| Asia | 19.9 | 35.0 | 20.7 | 15.2 | 7.3 | 1.8 |
| Australia | 20.8 | 69.2 | 9.6 | 0.5 | 0.0 | 0.0 |

**Figure S2.** Flowchart depicting the methodology and data considered in the study.

| **Variability** | | |
| --- | --- | --- |
|  | | |
| (i) **Monthly scale inter-annual variability of wet conditions** | | |
| (a) **NSPEI-1 (E-W)** | (b) **NSPEI-1 (S-W)** | (c) **NSPEI-1 (M-W)** |
|  |  |  |
| (d) **NSPEI-3 (E-W)** | (e) **NSPEI-3 (S-W)** | (f) **NSPEI-3 (M-W)** |
|  |  |  |
| (g) **NSPEI-6 (E-W)** | (h) **NSPEI-6 (S-W)** | (i) **NSPEI-6 (M-W)** |
|  |  |  |
| (j) **NSPEI-12 (E-W)** | (k) **NSPEI-12 (S-W)** | (l) **NSPEI-12 (M-W)** |
|  |  |  |
| (m) **NSPEI-24 (E-W)** | (n) **NSPEI-24 (S-W)** | (o) **NSPEI-24 (M-W)** |
|  |  |  |

|  | **Variability** | | | | | |
| --- | --- | --- | --- | --- | --- | --- |
|  |  | | | | | |
|  | (ii) **Monthly scale inter-annual variability of dry conditions** | | | | | |
|  | (a) **NSPEI-1 (M-D)** | | (b) **NSPEI-1 (S-D)** | | (c) **NSPEI-1 (E-D)** | |
|  |  | |  | |  | |
|  | (d) **NSPEI-3 (M-D)** | | (e) **SPEI-3 (S-D)** | | (f) **NSPEI-3 (E-D)** | |
|  |  | |  | |  | |
|  | (g) **NSPEI-6 (M-D)** | | (h) **NSPEI-6 (S-D)** | | (i) **NSPEI-6 (E-D)** | |
|  |  | |  | |  | |
|  | (j) **NSPEI-12 (M-D)** | | (k) **NSPEI-12 (S-D)** | | (l) **NSPEI-12 (E-D)** | |
|  |  | |  | |  | |
| (m) **NSPEI-24 (M-D)** | | (n) **NSPEI-24 (V-D)** | | (o) **NSPEI-24 (E-D)** | |  |
|  |  | |  | |  | |

| **Variability** | | |
| --- | --- | --- |
|  | | |
| (iii) **Monthly scale inter-annual variability of normal condition** | | |
| (a) **NSPEI-1 (N)** | (b) **NSPEI-3 (N)** | (c) **NSPEI-6 (N)** |
|  |  |  |
| (d) **NSPEI-12 (N)** | (e) **NSPEI-24 (N)** |  |
|  |  |  |

**Figure S3**. Monthly scale inter-annual variability (SVI_ME_) of (i) wet (W), (ii) dry (D), and (iii) normal (N) conditions for the period 1901 to 2019. In the notation NSPEI-*M*, *M* indicates the accumulation period in months. Extreme, severe, and moderate-intensity levels of wet conditions are denoted by E-W, S-W, M-W, respectively. The corresponding intensity levels in the case of dry conditions are denoted by E-D, S-D, M-D, respectively.

| **Variability** | | |
| --- | --- | --- |
|  | | |
| (i) **Inter-annual variability of wet conditions in the spring season** | | |
| (a) **NSPEI-1 (E-W)** | (b) **NSPEI-1 (S-W)** | (c) **NSPEI-1 (M-W)** |
|  |  |  |
| (d) **NSPEI-3 (E-W)** | (e) **NSPEI-3 (S-W)** | (f) **NSPEI-3 (M-W)** |
|  |  |  |
| (g) **NSPEI-6 (E-W)** | (h) **NSPEI-6 (S-W)** | (i) **NSPEI-6 (M-W)** |
|  |  |  |
| (j) **NSPEI-12 (E-W)** | (k) **NSPEI-12 (S-W)** | (l) **NSPEI-12 (M-W)** |
|  |  |  |
| (m) **NSPEI-24 (E-W)** | (n) **NSPEI-24 (S-W)** | (o) **NSPEI-24 (M-W)** |
|  |  |  |

| **Variability** | | | | |
| --- | --- | --- | --- | --- |
|  | | | | |
| (ii) **Inter-annual variability of wet conditions in the summer season** | | | | |
| (a) **NSPEI-1 (E-W)** | | (b) **NSPEI-1 (S-W)** | (c) **NSPEI-1 (M-W)** | |
|  | |  |  | |
| (d) **NSPEI-3 (E-W)** | | (e) **NSPEI-3 (S-W)** | (f) **NSPEI-3 (M-W)** | |
|  | |  |  | |
| (g) **NSPEI-6 (E-W)** | (h) **NSPEI-6 (S-W)** | | | (i) **NSPEI-6 (M-W)** |
|  |  | | |  |
| (j) **NSPEI-12 (E-W)** | (k) **NSPEI-12 (S-W)** | | | (l) **NSPEI-12 (M-W)** |
|  |  | | |  |
| (m) **NSPEI-24 (E-W)** | (n) **NSPEI-24 (S-W)** | | | (o) **NSPEI-24 (M-W)** |
|  |  | | |  |

| **Variability** | | |
| --- | --- | --- |
|  | | |
| (iii) **Inter-annual variability of wet conditions in the autumn season** | | |
| (a) **NSPEI-1 (E-W)** | (b) **NSPEI-1 (S-W)** | (c) **NSPEI-1 (M-W)** |
|  |  |  |
| (d) **NSPEI-3 (E-W)** | (e) **NSPEI-3 (S-W)** | (f) **NSPEI-3 (M-W)** |
|  |  |  |
| (g) **NSPEI-6 (E-W)** | (h) **NSPEI-6 (S-W)** | (i) **NSPEI-6 (M-W)** |
|  |  |  |
| (j) **NSPEI-12 (E-W)** | (k) **NSPEI-12 (S-W)** | (l) **NSPEI-12 (M-W)** |
|  |  |  |
| (m) **NSPEI-24 (E-W)** | (n) **NSPEI-24 (S-W)** | (o) **NSPEI-24 (M-W)** |
|  |  |  |

| **Variability** | | | |
| --- | --- | --- | --- |
|  | | | |
| (iv) **Inter-annual variability of wet conditions in the winter season** | | | |
| (a) **NSPEI-1 (E-W)** | (b) **NSPEI-1 (S-W)** | | (c) **NSPEI-1 (M-W)** |
|  |  | |  |
| (d) **NSPEI-3 (E-W)** | (e) **NSPEI-3 (S-W)** | | (f) **NSPEI-3 (M-W)** |
|  |  | |  |
| (g) **NSPEI-6 (E-W)** | | (h) **NSPEI-6 (S-W)** | (i) **NSPEI-6 (M-W)** |
|  | |  |  |
| (j) **NSPEI-12 (E-W)** | | (k) **NSPEI-12 (S-W)** | (l) **NSPEI-12 (M-W)** |
|  | |  |  |
| (m) **NSPEI-24 (E-W)** | | (n) **NSPEI-24 (S-W)** | (o) **NSPEI-24 (M-W)** |
|  | |  |  |

**Figure S4.** Inter-annual variability (SVI_ME_) of wet conditions in different seasons ((i) spring, (ii) summer, (iii) autumn and (iv) winter during the period 1901 to 2019. In the notation NSPEI-*M*, *M* indicates the accumulation period in months. Extreme, severe and moderate-intensity levels of wet conditions are denoted by E-W, S-W, M-W, respectively.

| **Variability** | | |
| --- | --- | --- |
|  | | |
| (i) **Inter-annual variability of dry conditions in the spring season** | | |
| (a) **NSPEI-1 (M-D)** | (b) **NSPEI-1 (S-D)** | (c) **NSPEI-1 (E-D)** |
|  |  |  |
| (d) **NSPEI-3 (M-D)** | (e) **NSPEI-3 (S-D)** | (f) **NSPEI-3 (E-D)** |
|  |  |  |
| (g) **NSPEI-6 (M-D)** | (h) **NSPEI-6 (S-D)** | (i) **NSPEI-6 (E-D)** |
|  |  |  |
| (j) **NSPEI-12 (M-D)** | (k) **NSPEI-12 (S-D)** | (l) **NSPEI-12 (E-D)** |
|  |  |  |
| (m) **NSPEI-24 (M-D)** | (n) **NSPEI-24 (S-D)** | (o) **NSPEI-24 (E-D)** |
|  |  |  |

| **Variability** | | |
| --- | --- | --- |
|  | | |
| (ii) **Inter-annual variability of dry conditions in the summer season** | | |
| (a) **NSPEI-1 (M-D)** | (b) **NSPEI-1 (S-D)** | (c) **NSPEI-1 (E-D)** |
|  |  |  |
| (d) **NSPEI-3 (M-D)** | (e) **NSPEI-3 (S-D)** | (f) **NSPEI-3 (E-D)** |
|  |  |  |
| (a) **NSPEI-6 (M-D)** | (b) **NSPEI-6 (S-D)** | (c) **NSPEI-6 (E-D)** |
|  |  |  |
| (a) **NSPEI-12 (M-D)** | (b) **NSPEI-12 (S-D)** | (c) **NSPEI-12 (E-D)** |
|  |  |  |
| (d) **NSPEI-24 (M-D)** | (e) **NSPEI-24 (S-D)** | (f) **NSPEI-24 (E-D)** |
|  |  |  |

| **Variability** | | |
| --- | --- | --- |
|  | | |
| (iii) **Inter-annual variability of dry conditions in the autumn season** | | |
| (a) **NSPEI-1 (M-D)** | (b) **NSPEI-1 (S-D)** | (c) **NSPEI-1 (E-D)** |
|  |  |  |
| (d) **NSPEI-3 (M-D)** | (e) **NSPEI-3 (S-D)** | (f) **NSPEI-3 (E-D)** |
|  |  |  |
| (g) **NSPEI-6 (M-D)** | (h) **NSPEI-6 (S-D)** | (i) **NSPEI-6 (E-D)** |
|  |  |  |
| (a) **NSPEI-12 (M-D)** | (b) **NSPEI-12 (S-D)** | (c) **NSPEI-12 (E-D)** |
|  |  |  |
| (d) **NSPEI-24 (M-D)** | (e) **NSPEI-24 (S-D)** | (f) **NSPEI-24 (E-D)** |
|  |  |  |

| **Variability** | | | | |
| --- | --- | --- | --- | --- |
| (iv) **Inter-annual variability of dry conditions in the winter season** | | | | |
| (a) **NSPEI-1 (M-D)** | (b) **NSPEI-1 (S-D)** | | (c) **NSPEI-1 (E-D)** | |
|  |  | |  | |
| (d) **NSPEI-3 (M-D)** | (e) **NSPEI-3 (S-D)** | | (f) **NSPEI-3 (E-D)** | |
|  |  | |  | |
| (g) **NSPEI-6 (M-D)** | | (h) **NSPEI-6 (S-D)** | (i) **NSPEI-6 (E-D)** |  |
|  |  | |  | |
| (a) **NSPEI-12 (M-D)** | | (b) **NSPEI-12 (S-D)** | (c) **NSPEI-12 (E-D)** |  |
|  |  | |  | |
| (d) **NSPEI-24 (M-D)** | | (e) **NSPEI-24 (S-D)** | (f) **NSPEI-24 (E-D)** |  |
|  |  | |  | |

**Figure S5.** Inter-annual variability of the variability (SVI_ME_) of dry conditions for different seasons ((i) spring, (ii) summer, (iii) autumn and (iv) winter) during the period 1901 to 2019. In the notation NSPEI-*M*, *M* indicates the accumulation period in months. Extreme, severe and moderate-intensity levels of dry conditions are denoted by E-D, S-D, M-D, respectively.

| **Variability** | | |
| --- | --- | --- |
| (i) **Variability of normal condition in the spring season** | | |
| (a) NSPEI-1 (N) | (b) NSPEI-3 (N) | (c) NSPEI-6 (N) |
|  |  |  |
| (d) NSPEI-12 (N) | (e) NSPEI-24 (N) |  |
|  |  |  |
| (ii) **Variability of normal condition in the summer season** | | |
| NSPEI-1 (N) | NSPEI-3 (N) | NSPEI-6 (N) |
|  |  |  |
| NSPEI-12 (N) | NSPEI-24 (N) |  |
|  |  |  |
| (iii) **Variability of normal condition in the autumn season** | | |
| (a) NSPEI-1 (N) | (b) NSPEI-3 (N) | (c) NSPEI-6 (N) |
|  |  |  |
| (d) NSPEI-12 (N) | (e) NSPEI-12 (N) |  |
|  |  |  |

| (iv) **Variability of normal condition in the winter season** | | |
| --- | --- | --- |
| (a) NSPEI-1 (N) | (b) NSPEI-3 (N) | (c) NSPEI-6 (N) |
|  |  |  |
| (d) NSPEI-12 (N) | (e) NSPEI-24 (N) |  |
|  |  |  |

**Figure S6:** Inter-annual variability (SVI_ME_) of normal (N) condition for (i) spring (ii) summer, (iii) autumn and (iv) winter season for the period 1901-2019.

(i) **Inter-annual variability of wet conditions for different seasons across North America**

(ii) **Inter-annual variability of wet conditions for different seasons across South America**

(iii) **Inter-annual variability of wet conditions for different seasons across Africa**

(iv) **Inter-annual variability of wet conditions for different seasons across Europe**

(v) **Inter-annual variability of wet conditions for different seasons across Asia**

(vi) **Inter-annual variability of wet conditions for different seasons across Australia**

**Figure S7.** Seasonal scale inter-annual variability (SVI_ME_) of wet conditions for different LULC classes for (i) North America (ii) South America (iii) Africa (iv) Europe (v) Asia (vi) Australia.

(i) **Inter-annual variability of dry conditions for different seasons across North America**

(ii) **Inter-annual variability of dry conditions for different seasons across South America**

(iii) **Inter-annual variability of dry conditions for different seasons across Africa**

(iv) **Inter-annual variability of dry conditions for different seasons across Europe**

(v) **Inter-annual variability of dry conditions for different seasons across Asia**

(vi) **Inter-annual variability of dry conditions for different seasons across Australia**

**Figure S8.** Seasonal scale inter-annual variability (SVI_ME_) of dry conditions for different LULC classes for (i) North America (ii) South America (iii) Africa (iv) Europe (v) Asia (vi) Australia

**Variability ranges**

(i) **Monthly scale inter-annual variability of extreme wet condition with elevation**

(a) **NSPEI-1**

(b) **NSPEI-3**

(c) **NSPEI-6**

(d) **NSPEI-12**

(e) **NSPEI-24**

(ii) **Monthly scale inter-annual variability of severe wet condition with elevation**

(a) **NSPEI-1**

(b) **NSPEI-3**

(c) **NSPEI-6**

(d) **NSPEI-12**

(e) **NSPEI-24**

(iii) **Monthly scale inter-annual variability of moderate wet condition with elevation**

(a) **NSPEI-1**

(b) **NSPEI-3**

(c) **NSPEI-6**

(d) **NSPEI-12**

(e) **NSPEI-24**

(iv) **Monthly scale inter-annual variability of normal condition with elevation**

(a) **NSPEI-1**

(b) **NSPEI-3**

(c) **NSPEI-6**

(d) **NSPEI-12**

(e) **NSPEI-24**

(v) **Monthly scale inter-annual variability of moderate dry condition elevation**

(a) **NSPEI-1**

(b) **NSPEI-3**

(c) **NSPEI-6**

(d) **NSPEI-12**

(e) **NSPEI-24**

(vi) **Monthly scale inter-annual variability of severe dry condition with elevation**

(a) **NSPEI-1**

(b) **NSPEI-3**

(c) **NSPEI-6**

(d) **NSPEI-12**

(e) **NSPEI-24**

(vii) **Monthly scale inter-annual variability of extreme dry condition with elevation**

(a) **NSPEI-1**

(b) **NSPEI-3**

(c) **NSPEI-6**

(d) **NSPEI-12**

(e) **NSPEI-24**

**Figure S9**. Monthly scale inter-annual variability (SVI_AE_) in wet conditions (i, ii, iii), normal condition (iv) and dry conditions (v, vi) for different elevation ranges in various continents. In the notation NSPEI-*M*, *M* indicates the accumulation period (AP) in months.

| (i) **Trend in intra-annual variability of months in wet conditions** | | |
| --- | --- | --- |
| (a) NSPEI-1 (E-W) | (b) NSPEI-1 (S-W) | (c) NSPEI-1 (M-W) |
|  |  |  |
| (d) NSPEI-3 (E-W) | (e) NSPEI-3 (S-W) | (f) NSPEI-3 (M-W) |
|  |  |  |
| (g) NSPEI-6 (E-W) | (h) NSPEI-6 (S-W) | (i) NSPEI-6 (M-W) |
|  |  |  |
| (j) NSPEI-12 (E-W) | (k) NSPEI-12 (S-W) | (l) NSPEI-12 (M-W) |
|  |  |  |
| (m) NSPEI-24 (E-W) | (n) NSPEI-24 (S-W) | (o) NSPEI-24 (M-W) |
|  |  |  |

| (ii) **Trend in the frequency of months in wet conditions** | | |
| --- | --- | --- |
| (a) NSPEI-1 (E-W) | (b) NSPEI-1 (S-W) | (c) NSPEI-1 (M-W) |
|  |  |  |

| (d) NSPEI-3 (E-W) | (e) NSPEI-3 (S-W) | (f) NSPEI-3 (M-W) |
| --- | --- | --- |
|  |  |  |
| (a) NSPEI-6 (E-W) | (b) NSPEI-6 (S-W) | (c) NSPEI-6 (M-W) |
|  |  |  |
| (d) NSPEI-12 (E-W) | (e) NSPEI-12 (S-W) | (f) NSPEI-12 (M-W) |
|  |  |  |
| (g) NSPEI-24 (E-W) | (h) NSPEI-24 (S-W) | (i) NSPEI-24 (M-W) |
|  |  |  |

| (iii) **Trend in intra-annual variability of months in dry conditions** | | |
| --- | --- | --- |
| (a) NSPEI-1 (E-D) | (b) NSPEI-1 (S-D) | (c) NSPEI-1 (M-D) |
|  |  |  |
| (d) NSPEI-3 (E-D) | (e) NSPEI-3 (S-D) | (f) NSPEI-3 (M-D) |
|  |  |  |
| (g) NSPEI-6 (E-D) | (h) NSPEI-6 (S-D) | (i) NSPEI-6 (M-D) |
|  |  |  |
| (j) NSPEI-12 (E-D) | (k) NSPEI-12 (S-D) | (l) NSPEI-12 (M-D) |
|  |  |  |
| (m) NSPEI-24 (E-D) | (n) NSPEI-24 (S-D) | (o) NSPEI-24 (M-D) |
|  |  |  |

| (iv) **Trend in monthly scale frequency of dry months** | | |
| --- | --- | --- |
| (a) NSPEI-1 (E-D) | (b) NSPEI-1 (S-D) | (c) NSPEI-1 (M-D) |
|  |  |  |
| (d) NSPEI-3 (E-D) | (e) NSPEI-3 (S-D) | (f) NSPEI-3 (M-D) |
|  |  |  |
| (g) NSPEI-6 (E-D) | (h) NSPEI-6 (S-D) | (i) NSPEI-6 (M-D) |
|  |  |  |
| (j) NSPEI-12 (E-D) | (k) NSPEI-12 (S-D) | (l) NSPEI-12 (M-D) |
|  |  |  |
| (m) NSPEI-24 (E-D) | (n) NSPEI-24 (S-D) | (o) NSPEI-24 (M-D) |
|  |  |  |

| (v) **Trend in intra-annual variability of months in normal condition** | | |
| --- | --- | --- |
| (a) NSPEI-1 (N) | (b) NSPEI-3 (N) | (c) NSPEI-6 (N) |
|  |  |  |
| (d) NSPEI-12 (N) | (e) NSPEI-24 (N) |  |
|  |  |  |

| (vi) **The trend in frequency of months in normal condition** | | |
| --- | --- | --- |
| (a) NSPEI-1 (N) | (b) NSPEI-3 (N) | (c) NSPEI-6 (N) |
|  |  |  |
| (d) NSPEI-12 (N) | (e) NSPEI-24 (N) |  |
|  |  |  |
|  | | |

**Figure S10.** Trends in intra-annual variability (SVI_AE_) and frequency/count of months in wet (W) conditions (i, ii), dry (D) conditions (iii, iv) and normal (D) conditions (v, vi) identified corresponding to different accumulation periods in analysis with Mann-Kendall (MK) trend test considering 5% significance level.

**Variability**

(i) NSPEI-1

(ii) NSPEI-3

(iii) NSPEI-6

(iv) NSPEI-12

(v) NSPEI-24

**Figure S11.** Decadal scale variability (SVI_DE_) of moderate wet (M-W) conditions across different accumulation periods.

**Variability**

(vi) NSPEI-1

(vii) NSPEI-3

(viii) NSPEI-6

(ix) NSPEI-12

(x) NSPEI-24

**Figure S12.** Decadal scale variability (SVI_DE_) of severe wet (S-W) conditions across different accumulation periods.

**Variability**

(j) NSPEI-1

(ii) NSPEI-3

(v) NSPEI-6

(vi) NSPEI-12

(v) NSPEI-24

**Figure S13.** Decadal scale variability (SVI_DE_) of extreme wet (E-W) conditions across different accumulation periods.

**Variability**

(i) NSPEI-1

(ii) NSPEI-3

(iii) NSPEI-6

(iv) NSPEI-12

(v) NSPEI-24

**Figure S14.** Decadal scale variability (SVI_DE_) of moderately dry (M-D) conditions across different accumulation periods.

**Variability**

(i) NSPEI-1

(ii) NSPEI-3

(iii) NSPEI-6

(iv) NSPEI-12

(v) NSPEI-24

**Figure S15.** Decadal scale variability (SVI_DE_) of severe dry (S-D) conditions across different accumulation periods.

**Variability**

(i) NSPEI-1

(ii) NSPEI-3

(iii) NSPEI-6

(iv) NSPEI-12

(v) NSPEI-24

**Figure S16.** Decadal scale variability (SVI_DE_) of extreme dry (E-D) conditions across different accumulation periods.
